# Genetic surveillance shows spread of ACT resistance during period of malaria decline in Vietnam (2018-2020)

**DOI:** 10.3389/fgene.2024.1478706

**Published:** 2024-12-02

**Authors:** Johanna Helena Kattenberg, Mathijs Mutsaers, Van Hong Nguyen, Thi Hong Ngoc Nguyen, Arlette Umugwaneza, Maria Lara-Escandell, Xuan Xa Nguyen, Thi Huong Binh Nguyen, Anna Rosanas-Urgell

**Affiliations:** ^1^ Biomedical Sciences Department, Institute of Tropical Medicine, Antwerp, Belgium; ^2^ Department of Clinical Research, National Institute of Malariology, Parasitology and Entomology, Hanoi, Vietnam; ^3^ Department of Molecular Biology, National Institute of Malariology, Parasitology and Entomology, Hanoi, Vietnam; ^4^ Regional Artemisinin Initiative, RAI project, National Institute of Malariology, Parasitology and Entomology, Hanoi, Vietnam

**Keywords:** Plasmodium falciparum, malaria molecular surveillance, drug resistance, Vietnam, artemisinin resistance, piperaquine resistance, next-generation sequencing

## Abstract

**Introduction:**

Vietnam’s goal to eliminate malaria by 2030 is challenged by the further spread of drug-resistant *Plasmodium falciparum* malaria to key antimalarials, particularly dihydroartemisinin-piperaquine (DHA-PPQ).

**Methods:**

The custom targeted NGS amplicon sequencing assay, AmpliSeq Pf Vietnam v2, targeting drug resistance, population genetic- and other markers, was applied to detect genetic diversity and resistance profiles in samples from 8 provinces in Vietnam (n = 354), in a period of steep decline of incidence (2018–2020). Variants in 14 putative resistance genes, including *P. falciparum Kelch 13 (PfK13)* and *P. falciparum chloroquine resistance transporter (Pfcrt)*, were analyzed and within-country parasite diversity was evaluated. Other targets included KEL1-lineage markers and diagnostic markers of *Pfhrp2/3*.

**Results:**

A concerning level of DHA-PPQ resistance was detected. The C580Y mutation in *PfK13* was found in nearly 80% of recent samples, a significant rise from previous data. Vietnam has experienced a significant challenge with the spread of DHA-PPQ resistant malaria parasites, particularly in the provinces of Binh Phuoc and Gia Lai. Resistance spread to high levels in Binh Thuan prior to the country-wide treatment policy change from DHA-PPQ to pyronadine-artesunate (PA). A complex picture of PPQ-resistance dynamics was observed, with an increase of PPQ-resistance associated *Pfcrt* mutations, indicating an evolutionary response to antimalarial pressure. Additionally, the compensatory mutation C258W in *Pfcrt*, which increases chloroquine (CQ) resistance while reversing PPQ resistance, is emerging in Gia Lai following the adoption of PA as the first-line treatment. This study found high levels of multidrug resistance, with over 70% of parasites in 6 out of 8 provinces showing significant sulfadoxine-pyrimethamine (SP) resistance and widespread chloroquine-resistant *Pfcrt* haplotypes. We also report an absence of *P. falciparum histidine rich protein 2 and 3 (Pfhrp2/3)* gene deletions, ensuring the continued reliability of HRP2/3-based rapid diagnostic tests. *P. falciparum* populations in Vietnam are becoming more isolated, with clonal populations showing high geographical clustering by province. The central highlands, particularly Gia Lai province, have the highest residual malaria burden but exhibit low diversity and clonal populations, likely due to the pressures from the antimalarial drugs and targeted national malaria control program (NMCP) efforts.

**Discussion:**

In conclusion, examining a broad panel of full-length resistance genes and SNPs provided high-resolution insights into genetic diversity and resistance evolution in Vietnam, offering valuable information to inform local treatment and intervention strategies.

## Introduction

Over the past decade, Vietnam has seen a significant reduction in malaria incidence and mortality, leading to the successful elimination of malaria in 42 out of 63 provinces in 2022 (National Institute of Malariology and Parasitology and Entomology, 2022). The country is aiming to eliminate malaria by 2030. However, the emergence and spread of *Plasmodium falciparum* resistance to artemisinin-based combination therapies (ACT), among other therapies, poses a significant challenge for countries in the Greater Mekong Subregion (World Health Organization, 2022). Efficient tools for monitoring drug resistance are essential to support national malaria control programs (NMCP), particularly beyond the scope of resource-intensive therapeutic efficacy studies (TES). This necessity is even more critical in countries with low endemicity and those progressing towards elimination, where the decreasing number of cases makes TES logistically and financially challenging, as it becomes increasingly difficult to enroll sufficient patients to meet the required sample sizes. Recent advancements in genomic sequencing have made the technology more affordable and time-efficient, allowing researchers to monitor molecular markers using simple benchtop sequencers. Although surveillance of molecular (or genotypic) markers cannot confirm individual treatment outcomes, their rise often precedes treatment failures in a population, making it an early warning system prioritized by the WHO (World Health Organization) (World Health Organization, 2009; World Health Organization, 2018). As a result, these sequencing approaches provide valuable insights into both resistance and population genetics and can expand the geographic coverage of surveillance without facing the complex challenges posed by clinical trials.

This article focusses on the molecular surveillance of malaria in the provinces in Vietnam with the highest burden of residual malaria. Samples were collected at the North Central Coast, Central Highlands, South Central Coast and Southeast Region; geographical zones characterized by hilly and forested areas with persistent malaria endemicity. Together, these regions accounted for 81% and 97% of cases in respectively 2016 and 2019. At start of collection in 2018, Vietnam recorded 2,858 cases of *P. falciparum* annually. By 2020, it dropped to 825 cases and continued to decrease to 455 cases in 2022 (UCSF, 2021). Despite this strong progress, resistance continues to be a major concern hampering the last mile towards elimination. Delayed parasite clearance was first detected in 2009 after treatment with the first-line therapy of dihydroartemisinin-piperaquine (DHA-PPQ) in Binh Phuoc (Tran et al., 2004). Since then, studies showed an increase in artemisinin (ART) and piperaquine (PPQ) resistance, forcing NMCPs to update the first-line treatment guidelines in the affected provinces (Tran et al., 2004; Rovira-Vallbona et al., 2020; Thanh et al., 2017; Van Der Pluijm et al., 2019; Phong et al., 2019; Thriemer et al., 2014; Vietnam Ministry of Health, 2020). Nevertheless, no changes to the guidelines were implemented until 2017, with a first clinical trial starting in 2017–2019 to assess the alternative therapy of pyronaridine-artesunate, branded Pyramax^®^ (pyronadine-artesunate, PA) (UCSF, 2021; Manh et al., 2021; Quang Bui et al., 2020). PA was officially introduced in 2020 as an alternative treatment regimen in Vietnam for regions where DHA-PPQ treatment failure rates exceeded 10% (Cao et al., 2023).

At the time of writing, 13 non-synonymous mutations in the *P*. *falciparum Kelch 13* (*PfK13*) propellor region have been validated as artemisinin partial resistance markers according to the WHO, of which C580Y, R593T, Y493H, P553L and I543T are established in Vietnam and the wider Greater Mekong Subregion (GMS) (World Health Organization, 2018; Thanh et al., 2017; Rovira-Vallbona et al., 2023). Several non-*PfK13* genes exist that are likely to contribute to ACT resistance, such as *Pfcoronin*, *P*. *falciparum ubiquitin carboxyl-terminal hydrolase 1 (Pfubp1)* and *P*. *falciparum AP-2 complex subunit mu* (*Pfap2-mu)*, with few validated markers and background mutations (Demas et al., 2018; Henrici et al., 2020; Stokes et al., 2021; Sutherland et al., 2021; Ajibaye et al., 2024). A recent publication by Rovira-Vallbona et al. (2023) on molecular surveillance in Vietnam (2000-2016) indicated a 48.1% prevalence of *PfK13* validated markers (Rovira-Vallbona et al., 2023). These markers were detected in samples from as early as 2000, with a steady increase throughout the studied period, particularly in the Central Highlands bordering Cambodia. Moreover, a shift was observed from I543T to C580Y becoming the predominant marker, but with remarkable lower prevalence in the coastal provinces. This aligned with regional differences in treatment efficacy, where coastal provinces had fewer reports of treatment failure. The effectiveness of ACT therapies, however, remained high in most parts, because they are administered in combination with a partner drug. Notably, this is not the case for the whole of Vietnam, where co-emergence of resistance for the partner drug PPQ was previously observed in the KEL1/PLA1 lineage, threatening malaria elimination efforts (Rovira-Vallbona et al., 2023; Jacob et al., 2021).

Copy number variations (CNVs) of *P. falciparum plasmepsin-2* (*Pfpm2*) and *plasmepsin-3* have been strongly associated with reduced *in vitro* susceptibility to PPQ and reduced treatment efficacy of DHA-PPQ for uncomplicated *P. falciparum* malaria (Van Der Pluijm et al., 2019; Qidwai, 2020; Witkowski et al., 2017; Imwong et al., 2017; Ross et al., 2018; Hamilton et al., 2019). The KEL1/PLA1 lineage, highly prevalent in Binh Phuoc, carries a combination of CNVs of plasmepsin-2 in combination with the *PfK13* artemisinin resistant (ART-R) marker C580Y, that led to parasites acquiring increased tolerance to DHA-PPQ, and authorities to shift first-line therapy to PA in affected regions (Thanh et al., 2017). More recently, several emerging markers for PPQ resistance have been identified in the gene *P*. *falciparum chloroquine resistance transporter (Pfcrt)* (T93S, H97Y, F145I, I218F, M343L, C350R and G353V) (World Health Organization, 2018; Van Der Pluijm et al., 2019; Ross et al., 2018; Hamilton et al., 2019). Analyzing these CNVs and other validated markers for former, first-line and alternative therapies in the *P. falciparum* parasite population in Vietnam continues to be essential to assess the spread, prevalence and emergence of resistance.

In regions where former therapies are regaining efficiency, they may again play a role in the fight against malaria (Huong et al., 2003; Frosch et al., 2014; Asare et al., 2021). While chloroquine (CQ) is no longer used to treat *P. falciparum* due to high resistance levels in the past, it remains the first-line treatment for uncomplicated malaria caused by *P. vivax*, a species that is co-endemic in the region. Since 2023, PA has been added as an alternative first line treatment for *Plasmodium vivax* in the guidelines. In *P. falciparum*, resistance is attributed to a codon change in *Pfcrt* (K76T) and nearby mutations in codons 73-76, constructing the resistant CVIET and CVIDT haplotypes (Jacob et al., 2021; Fidock et al., 2000). These haplotypes are highly prevalent in the GMS and can also be associated with lowered susceptibility to PPQ, mefloquine (MQ), and lumefantrine (LUM) when carrying the Y184F background mutation in *P*. *falciparum multidrug resistance protein 1 (Pfmdr1)*, a marker validated to confer CQ resistance (Veiga et al., 2016). Also, the reintroduction of sulfadoxine-pyrimethamine (SP) is explored as a long-lasting component in ACT in Vietnam. In the late 1990s, *in vivo* SP resistance encountered in patients was 30%–80% according to local reports (Huong et al., 2003; Huong et al., 2001). Here, mutations in *Pfdhps* and *Pfdhfr* are at the root, the latter conferring low or high resistance levels depending on the accumulation of mutations (World Health Organization, 2018). Quadruple and triple mutants characterized by codon changes N51I, C59R, S108N and I164L in *Pfdhfr* and S436A/F, A437G, K540E, A581G and A613T/S in *Pfdhps* are linked to high pyrimethamine (PYR) and sulfadoxine (SULF) resistance, respectively, whereas single or double mutants at these positions confer low to moderate resistance levels. More recently, very highly SP-variants have been circulating with quintuple and sextuple mutants in these genes.

Using a custom targeted NGS amplicon sequencing assay for *P. falciparum* (NGS Pf AmpliSeq v2 Vietnam assay), in this study we amplify 14 putative drug resistance genes (Table 1), KEL1-lineage markers associated with ART-R, a custom designed 46 SNP-barcode for within-country analysis of parasite dynamics, and the diagnostic markers *Pfhrp2/3*. This wide range of targets includes all genes validated for ACT resistance by WHO as well as additional markers identified in literature review. This allows us to assess all (combination) therapies relevant to the Vietnamese context, with particular interest in ART, PPQ, CQ and SP. The 46 SNP barcode was designed using *P. falciparum* genomes from Vietnam, using a custom approach to select neutral SNPs that contribute to the within-country differentiation. By combining these SNPs for population genetic analysis with the phenotypic markers, the study sought to further explore the emergence, prevalence, and geographical spread of drug-resistant *P. falciparum* parasites, as well as the dynamics of the parasite population in a period of declining transmission due to intensified malaria control and elimination efforts in Vietnam. This will contribute to the development of more effective malaria control strategies, crucial in the last mile towards elimination.

**TABLE 1:** Genes of interest for antimalarial drug resistance targeted in the Pf AmpliSeq v2 Vietnam assay.

Gene ID	Chrom.	Gene name with resistance-associated SNP(s)	Drug associated with resistance^a^
PF3D7_0112200	1	multidrug resistance-associated protein 1 (*Pfmrp1*)	ART, MQ, LF
PF3D7_0104300	1	ubiquitin carboxyl-terminal hydrolase 1 (*Pfubp-1*)	ART
PF3D7_0417200	4	dihydrofolate reductase (*Pfdhfr*)	PYR, PG
PF3D7_0523000	5	multidrug resistance protein 1 (*Pfmdr1*)	CQ, PPQ, MQ, QN, HF, ART, AMQ, LF
PF3D7_0516500	5	major facilitator superfamily domain-containing protein (*Pfmfs1/mdt*)	DOX
PF3D7_0709000	7	chloroquine resistance transporter (*Pfcrt*)	CQ, PPQ, AMQ
PF3D7_0810800	8	dihydropteroate synthase (*Pfdhps*)	SULF
PF3D7_0813000	8	Kelch interacting protein 7 (*PfKIC7*)	ART
PF3D7_1025000	10	Eps15-like protein (*PfEps15/Formin2*)	ART
PF3D7_1218300	12	AP-2 complex subunit mu (*Pfap2-mu*)	ART, QN
PF3D7_1251200	12	PfCoronin	ART
PF3D7_1235400	12	tetQ family GTPase (*PftetQ*)	DOX
PF3D7_1343700	13	Kelch protein K13 (*PfK13*)	ART
PF3D7_1408000	14	Plasmepsin 2 (*Pfpm2*)	PPQ

^a^ The list of drugs to which resistance has been observed is non-exhaustive. Validated mutations are listed in the WHO report on antimalarial drug efficacy, Resistance and Response: 10 Years of Surveillance (2010–2019) (World Health Organization, 2018). In additionm the Infectious Diseases Data Observatory (IDDO) (2015): Artemisinin Molecular Surveyor, Infectious Diseases Data Observatory. https://www.iddo.org/wwarn/tracking-resistance/artemisinin-molecular-surveyor tracks *PfK13* ART-R and partner drug-markers. AMQ, amodiaquine; ART, artesunate; ATQ, atovaquone; CQ, chloroquine; CHROM, chromosome;DHA, dihydroartemisinin; DOX, doxycycline, HF, halofantrine; LF, lumefantrine; MQ, mefloquine; PG, proguanil; PPQ, piperaquine; PYR, pyrimethamine; SULF, sulfadoxine; QN, quinine.

## Methods

### Sample collection

Routine collections of dried blood spots (DBS) were conducted at selected sentinel site health centers (2018-2020) in the malaria-endemic provinces Binh Thuan, Binh Phuoc, Gia Lai, Khanh Hoa, Kon Tum, Lam Dong, Ninh Thuan, and Quang Tri to monitor uncomplicated malaria (*P. falciparum* and *P. vivax*) in Vietnam. In each province at least 1 sentinel site was chosen supporting several surrounding communities. In provinces with many malaria cases (such as Gia Lai, Khanh Hoa, Lam Dong), samples were collected at 2 sentinel sites, often one in a district hospital and one in a commune. Distribution of this collection depended heavily on the caseload in each province, with some provinces reporting a very low number of malaria cases (Table 2). Individuals between 7–60 years old living around the sentinel site clinic, visiting the clinic with suspected malaria were invited to take part in the surveillance. In addition, 11 samples from previous routine collections in 2015–2017 from Dak Nong (n = 3), Dak Lak (n = 3) and Khanh Hoa (n = 4) were collected for whole-genome sequencing (WGS) analysis for the barcode design, as there was no prior genomic data available from these sites, and one additional sample from Binh Phuoc included. DBS were collected from confirmed malaria cases (positive by microscopy and/or RDT (Rapid Diagnostic Test)) when informed consent had been given. Details of the *P. vivax* cases are described elsewhere (Kattenberg et al., 2022).

**TABLE 2:** Overview of samples included in Pf AmpliSeq v2 Vietnam analysis.

Province	N per province	year	N per year	% On total	% On province	NMCP recorded *Pf* cases	% Of NMCP cases genetically analyzed
Binh Phuoc	19	2018	9	2.5%	47.4%	728	1.2%
		2019	10	2.8%	52.6%	120	8.3%
		2020	0	0.0%	0.0%	23	0.0%
Binh Thuan	37	2018	8	2.3%	21.6%	29	27.6%
		2019	28	7.9%	75.7%	187	15.0%
		2020	1	0.3%	2.7%	42	2.4%
Gia Lai	231	2018	47	13.3%	20.3%	758	6.2%
		2019	78	22.0%	33.8%	1,296	6.0%
		2020	106	29.9%	45.9%	492	21.5%
Khanh Hoa	34	2018	31	8.8%	91.2%	85	36.5%
		2019	2	0.6%	5.9%	33	6.1%
		2020	1	0.3%	2.9%	5	20.0%
Kon Tum	7	2018	7	2.0%	100.0%	66	10.6%
		2019	0	0.0%	0.0%	15	0.0%
		2020	0	0.0%	0.0%	5	0.0%
Lam Dong	6	2018	6	1.7%	100.0%	28	21.4%
		2019	0	0.0%	0.0%	83	0.0%
		2020	0	0.0%	0.0%	19	0.0%
Ninh Thuan	9	2018	9	2.5%	100.0%	26	34.6%
		2019	0	0.0%	0.0%	44	0.0%
		2020	0	0.0%	0.0%	4	0.0%
Quang Tri	11	2018	0	0.0%	0.0%	65	0.0%
		2019	11	3.1%	100.0%	13	84.6%
		2020	0	0.0%	0.0%	3	0.0%
ALL	354						

**FIGURE 1:**
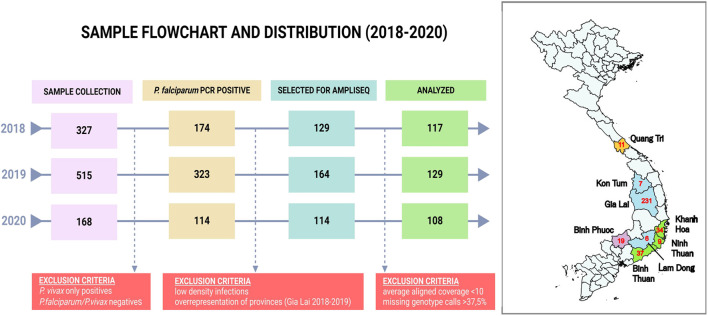
Sample flowchart and distribution by province in Vietnam. The flowchart indicates the sample selection procedure for analysis. RDT and LM positive *Plasmodium falciparum* and/or *Plasmodium vivax* samples (n = 1,010) were collected in provinces highlighted in the map. *Plasmodium vivax* PCR positive samples (n = 372) were analyzed in a separate study (Kattenberg et al., 2022). A small number of samples was negative for both species (n = 26, 2.5%), and are likely infections with minor species or contain PCR inhibitors. From the 611 *Plasmodium falciparum* PCR positive samples we selected 407 samples for sequencing based on parasite density (10 parasites/μl, determined after a first assessment of sequencing succes in 2018). In addition, not all samples from Gia Lai province were included due to the high number of samples from that province. Red numbers in the map indicate the number of samples analyzed in the Pf AmpliSeq v2 Vietnam assay from each province. Quang Tri is in the North Central Coast Region (orange). Kon Tum, Gia Lai and Lam Dong are in the Central Highlands Region (blue). Khanh Hoa, Ninh Thuan and Binh Thuan are in the South Central Coast Region (green), and Binh Phuoc is in the Southeast Region of Vietnam (purple). Map created using QGIS v3.40.0 with spatial data obtained from https://www.divs-gis.org/.

A total of 1010 DBS samples were available from sentinel site collections (Figure 1) and DNA was extracted using one 5 mm punch (2019 samples) or three 5 mm punches (all other DBS samples) of DBS using the QIAamp DNA Blood Mini Kit (Qiagen) with a final elution in 200 μL elution buffer from the kit. VarATS and 18S-qPCR (Hofmann et al., 2015) were performed to confirm diagnosis (Ct < 42) and 407 samples were included for library preparation (Figure 1).

### SNP selection for barcode design using WGS

To design a SNP barcode with in-country resolution in Vietnam, raw WGS data (FASTQ files) of *P. falciparum* isolates from Vietnam (2015-2017) generated in-house (n = 11) was combined with a VCF file previously generated using Vietnamese and Cambodian *P. falciparum* genomes (n = 262) from our lab that were externally sequenced as part of MalariaGEN SpotMalaria Project (https://www.malariagen.net/projects/SpotMalaria as described in (16). Newly generated sequencing data for the 11 isolates was deposited in SRA under project number PRJNA1156587, accession numbers are listed in Supplementary Table S1.

In house generated FASTQ files were aligned to the 3D7 reference genome version 41 from PlasmoDB [https://plasmodb.org/plasmo/app/downloads/release-41/Pfalciparum3D7/] and variants were called using GATK4 HaplotypeCaller and merged using bcftools (Danecek et al., 2021; Poplin et al., 2017). Variants were filtered on DP > 50, keeping only SNPs with at least 1 alternate allele (*i.e.*, removing SNPs that were homozygous reference in all samples or missing in all samples from the SpotMalaria dataset), resulting in 53,362 high quality SNPs.

Three samples were identified as outliers in principal component analysis (PCA) and removed from further analysis (SPT25139, SPT25147 and SPT25151). The remaining samples were used in a pipeline to select SNPs for the within-country barcode, in a similar procedure as the selection of SNPs for the *P. vivax* barcode for Vietnam (Kattenberg et al., 2022). Briefly, LD-pruning was performed iteratively using 500 bp windows, removing SNPs with pairwise LD > 0.2 via scikit-allel (Miles et al., 2024). Remaining SNPs were filtered for minor allele frequency (MAF) > 10%, and selectively neutral SNPs were retained with |Tajima’s D| > 0.5 (calculated in 500 bp windows), resulting in 638 SNPs. Subsequently, the contributions of the SNPs to geospatial genetic clusters were determined using discriminant analysis of principal components (DAPC) with province populations using adegenet package in R (Jombart et al., 2010). We conducted a parameter sweep for the DAPC varying the number of discriminant analysis (DA) clusters between 30-40, number of principal components between 25-40 and number of axes in the DA between 4-6 resulting in a list of 638 potential SNPs. Per chromosome, we selected 4-7 SNPs with loading contributions in the DAPC > 0.003 with the highest count in the DAPC parameter sweep to add to the design.

The Illumina Concierge team (Illumina, San Diego, United States) used DesignStudio software with the Pf3D7 reference genome to design amplicons for the new barcode and other selected markers. Amplicon design was successful for 46 SNPs. Based on recent literature research, we identified several new genes of potential interest for artemisinin resistance. Therefore, compared to our previous Pf Peru AmpliSeq panel, we targeted the additional genes *PfKIC7* (PF3D7_0813000) and *PfEps15/Formin 2* (PF3D7_1025000), both associated with *in vitro* resistance to ART (Henrici et al., 2020; Birnbaum et al., 2020; Kattenberg et al., 2023a). In addition, the targets *Pfmdt* (PF3D7_0516500) and *PftetQ* (PF3D7_1235400) were added, where CNVs in both genes have been associated with doxycycline resistance (Briolant et al., 2010; Gaillard et al., 2015). We no longer targeted the *cytB* gene and *23S rRNA* genes, as the mitochondrial and apicoplast templates are more predominant than nuclear targets and are overrepresented in the resulting sequences (Kattenberg et al., 2023a). In the amplicon design, full resistance associated genes were targeted (Table 1), as well as six KEL1-lineage markers (incl. *RAD5* and *arps10*), variable regions D1 and D2 in *PfamaI*, and *Pfhrp2* and *Pfhrp3*. The final designed panel for the Pf AmpliSeq v2 Vietnam included 265 amplicons, ranging in size from 115–379 bp (Supplementary Table S2).

### AmpliSeq library preparation and sequencing

Library preparation was done for each sample and control using custom primers (Pf AmpliSeq v2 Vietnam) and an AmpliSeq Library PLUS kit (Illumina), as previously described in a step-by-step laboratory protocol (Kattenberg et al., 2023b). Briefly, libraries were indexed (Illumina), purified using AMPure XP magnetic beads (Beckman Coulter) and quantified using Qubit v3 High sensitivity DNA kit (Invitrogen). Libraries were diluted to 2 nM with low Tris-EDTA buffer, and pooled (pooling 96–144 libraries per sequencing run). Denatured library pool (7 pM) was loaded on a MiSeq system (Illumina) for 2 × 300 paired-end sequencing (Miseq Reagent Kit v3, Illumina) with 5% PhiX spike-in (Illumina).

A set of DNA controls provided through BEI Resources was used to validate the detection resistant markers in *PfK13* (MRA-1241, MRA-1251, MRA-1255). The specificity of the assay was validated using extracted human malaria-negative blood, 3D7 reference wild type, D10 drug sensitive lab strain, Dd2 lab strain and mock Pf-Pv co-infections. Sensitivity of the assay was validated using a 3D7 dilution from 2,450 to 1,000 parasites/µL, followed by 1:10 dilutions up to 1 parasite/µL. Drug resistance variant calling, and population structuring was validated using 23 previously characterized samples from Cambodia and Vietnam that were genotyped using a TruSeq Custom Assay and SpotMalaria genetic report card (Rovira-Vallbona et al., 2020; Rovira-Vallbona et al., 2023).

### Bioinformatic data processing

FASTQ files were processed using an in-house bioinformatic pipeline published on GitHub (https://github.com/Ekattenberg/Plasmodium-AmpliSeq-Pipeline). Briefly, demultiplexed reads were trimmed (Trimmomatic) to remove adapter sequences and low-quality bases (Bolger et al., 2014). Trimmed reads were then aligned to the reference genome 3D7 (version 44, PlasmoDB) using Burrows-Wheeler aligner (Li and Durbin, 2009). Alignment statistics were generated with Picard’s tools and variants were called using GATK4 haplotypecaller generating a jointly-called VCF file with variants (SNPs and INDELs) detected in the targeted regions (Poplin et al., 2017; Picard Toolkit, 2019). Variants were hard filtered (QUAL>30, overall DP > 500, MQ > 50, QD > 1.0, SOR<4, ReadPosRankSum > −10.0, GT depth >5) and annotated with SnpEff (v4.3T) (Cingolani et al., 2012), resulting in 2,215 high quality genotypes (incl. all variant types, *e.g.*, SNPs and indels). Per locus filtered depth of coverage (format field DP in the VCF) was used to calculate the median depth of all loci per sample or per amplicon. Aligned coverage was calculated as the number of bases passed filter divided by the number of bases (67,503 bp) targeted in the Pf AmpliSeq v2 Vietnam.

Samples with good quality sequences that passed our inclusion criteria (average aligned coverage >10; and missing genotype calls <37.5%) were selected for further analysis. In total 354 samples were analyzed coming from Gia Lai (231/354), Binh Thuan (37/354), Khanh Hoa (34/354), Binh Phuoc (19/354), Quang Tri (11/354), Ninh Thuan (9/354), Kon Tum (7/354), (Lam Dong (6/354) (Table 2).

### Data analysis

We created a list of variants of interest (Supplementary Table S3) that included variants validated for resistance according to the WHO, WARNN meta-analysis and mutations in targeted genes reported in the literature as potentially associated with *P. falciparum* antimalarial resistance (World Health Organization, 2018; Rovira-Vallbona et al., 2023; Kattenberg et al., 2023a; Das et al., 2013). The list was supplemented with non-synonymous variants detected in the target genes that contributed to the variation in the DAPC. Haplotypes were created by combining genotypes of variants of interest.

Genetic diversity, expressed as expected heterozygosity (*He*), was calculated using polymorphic barcode (46 SNPs) genotypes from the VCF with the adegenet package in R (Jombart, 2008; Jombart and Ahmed, 2011). To compare the median of the genetic diversity parameters across different provinces, Kruskal-Wallis rank sum test was performed. For pairwise comparisons between groups, Wilcoxon test with Benjamini-Hochberg correction for multiple testing was used. Statistical tests were performed with the R package stats. P-values <0.05 were considered significant. PCA and DAPC with cross-validation was performed to infer population structure based on haplotypes across districts and years (Jombart et al., 2010). Associated allele loadings for the first four components in the DAPC were determined. Genetic differentiation, expressed as fixation index (F_ST_), was calculated using barcode SNP genotypes using the R package hierfstat (Goudet et al., 2022). Within-host infection complexity was assessed using within-sample F-statistic (Fws) with the R package moimix using all biallelic SNPs (n = 1,276) detected by the assay (Lee and Bahlo, 2016). Fws ≥0.95 was considered a proxy for a monoclonal infection as in Auburn et al. (2012).

To measure pairwise identity-by-descent (IBD) between samples, PED and MAP file formats were generated from VCF data using VCFtools (Danecek et al., 2011). The level of IBD-sharing was calculated employing the isoRelate package in R (Henden et al., 2018). We used all biallelic SNPs (n = 1,276) identified by the Pf AmpliSeq v2 Vietnam assay, applying filtering criteria of MAF = 0.001 and SNP and individual missingness thresholds (0.6 and 0.3, respectively), resulting in 952 SNPs for subsequent IBD analysis. Furthermore, IBD thresholds were set to include a minimum of 15 SNPs per segment and a segment length of 3,000 bp, aimed at mitigating false-positive calls using an error of 0.001. Network plots (at thresholds of 90%) were created using the igraph package in R to visualize the relatedness between samples and connectivity between districts as described before (Kattenberg et al., 2022; Kattenberg et al., 2024; Csárdi et al., 2024).

The read depth was extracted for each amplicon in each individual BAM file using samtools (selecting only reads with MQ ≥ 60) (Danecek et al., 2021). For this analysis we included only samples with mean read depth between 500 and 9,000 (306/354 samples). The read depths were normalized (creating values between 0 and 1) using the normalize function of the heatmaply package in R (Galili et al., 2018). Samples were clustered based on the amplicons of the target genes (*Pfpm2*, *Pfmdr1*, *Pfhrp2*, or *Pfhrp3*) and reference amplicons with little variation in depth (Supplementary Figure S1) and where possible on the same chromosome as the target gene. Hierarchical clustering was performed using the heatmaply package in R, based on Euclidean distances between read depth profiles of samples and the complete clustering method. Clustering patterns and normalized read depth were visually inspected in the resulting heatmaps. Individual sample *Pfpm2* amplifications were determined for samples belonging to two clusters that contained qPCR confirmed CNV in this gene and had clear increase in read depth compared to the reference amplicons.

### Ethics statement

The sample collections in Vietnam were approved by local ethical review boards at NIMPE and by agreement of the Ministry of Health in Vietnam. Individuals were included in this study only if they willingly signed informed consent that included an opt-in future-use clause. Secondary use of all samples was approved through the Institutional Review Board of the Institute of Tropical Medicine Antwerp (reference 1417/20).

## Results

We have developed a highly-multiplexed sequencing assay for cost-effective targeted deep sequencing of *P. falciparum* as described in detail in the methods section. This Pf AmpliSeq v2 Vietnam assay combines phenotypic and population genetic markers suitable for multiple genetic surveillance use cases demonstrated below. To improve the applicability and relevance of our targeted sequencing approach for molecular surveillance of *P. falciparum* in Vietnam, we designed a SNP barcode with in-country resolution in Vietnam.

### Design and *in silico* evaluation of the Pf Vietnam-barcode using WGS

**FIGURE 2:**
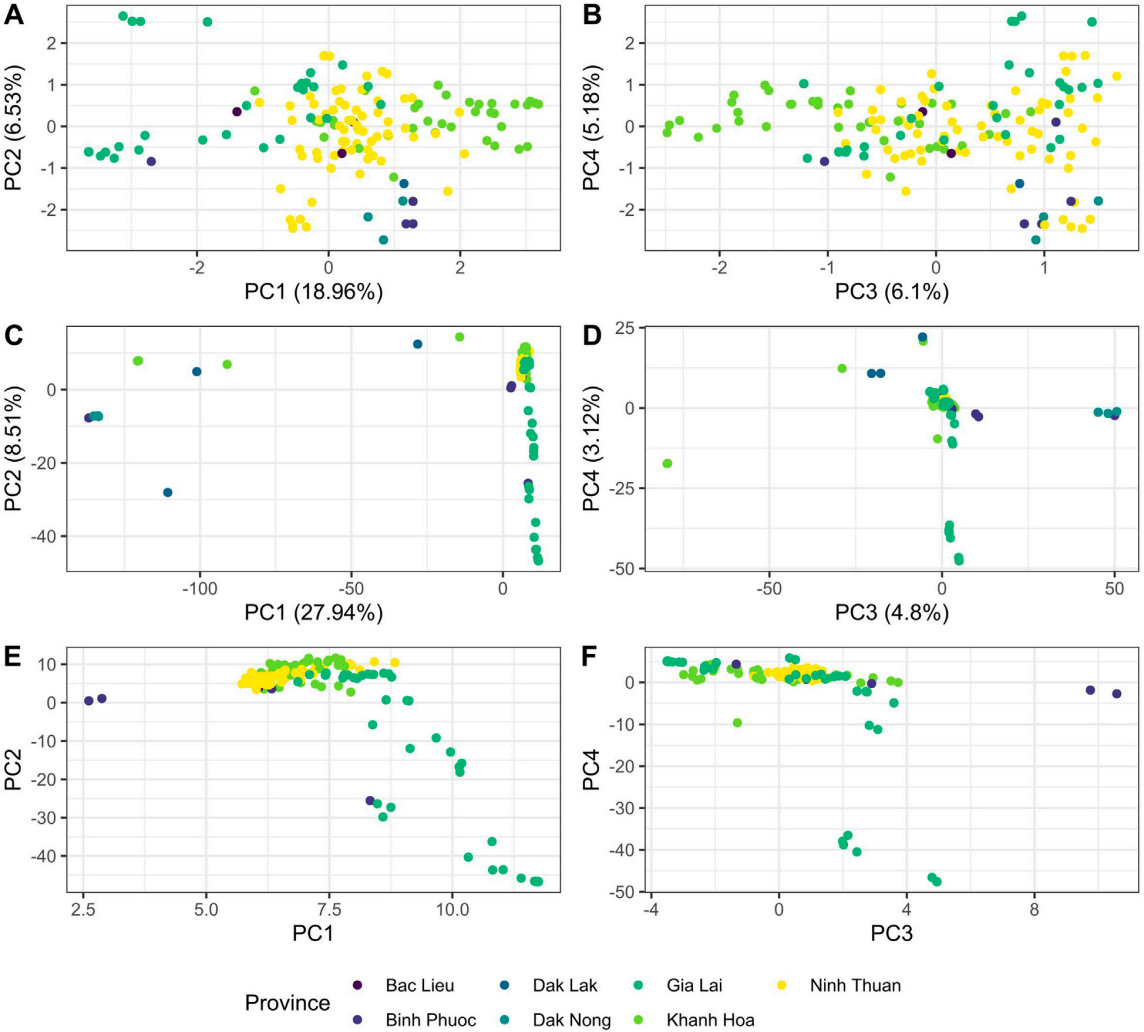
PCA analysis of *Plasmodium falciparum* genomes from Vietnam with different SNP subsets in WGS data. **(A, B)** PCA analysis was conducted with barcode SNPs only (n_loci_ = 46) in panels **(A)** and **(B)**. Panel **(A)** shows principal components 1 vs. 2, while panel B shows PC 3 vs. 4. In addition, PCA was performed with all biallelic SNPs [panels **(C–F)**] in the genomes (n = 44,553 SNPs). Panels C and E show PC 1 vs PC2 and panels D&F show PC 3 vs PC4. In Panels E and F PCA was performed with all biallelic SNPs, but with the strongest separating isolates from Dak Nong and Dak Lak were removed (PCA score <0 in PC1) to better observe the clustering patterns of the remaining samples from other provinces.

We designed a 46-SNP Pf Vietnam Barcode with in-country resolution, able to differentiate distinct *P. falciparum populations* in Vietnam based on the variability of allele frequencies between provinces. This barcode was developed using *P. falciparum* genomes from Vietnam and Cambodia (n = 273), where neutral SNPs were selected based on their contribution to geographically distinct genetic clusters in DAPC. The minor allele frequencies of all SNPs in the barcode varied across a range of 0.11–0.49, with a median value of 0.36 (Supplementary Table S4). The Pf Vietnam Barcode was able to differentiate the samples by province in the PCA analysis, although clusters where less pronounced than with all biallelic SNPs (n = 44,553 SNPs) in the WGS data (Figure 2). With all biallelic SNPs, the strongest differentiation was observed with the cluster of samples from Dak Nong and some samples from Dak Lak (along PC1 in Figure 2C), which also separated with the barcode SNPs (along PC2 in Figure 2A). In addition, strong differentiation was seen with part of the samples from Gia Lai with both the barcode and all biallelic SNPs (Figure 2A, E, F). The samples from Khanh Hoa and Ninh Thuan clustered close together but could still be differentiated at the resolution of the SNP barcode (Figures 2A, B).

#### Barcode and Pf AmpliSeq v2 Vietnam assay performance

The performance of the 46 SNP-barcode in the Pf AmpliSeq v2 Vietnam assay was tested in DBS samples collected in sentinel sites in 8 provinces in Vietnam with the highest burden of malaria in 2018–2020. Of the 407 samples processed, 354 passed our inclusion criteria of average depth of coverage above 10 and missing less than 37.5% genotype calls. Samples that did not pass inclusion thresholds had low parasite density and for future studies we advise a parasite density threshold of 10 parasites/µL (by VarATS qPCR) for inclusion in library preparation (Supplementary Figure S2).

Primer specificity was confirmed using uninfected human blood samples (n = 5) and *P. vivax* infected samples from Vietnam (from (34)) (n = 3) as negative controls. The few sequencing reads generated (median 15,645 reads) mapped predominantly outside the assay target regions (median 22.4% of reads aligned), but all aligned and called variants were below the filtering and inclusion thresholds for analysis.

All 46 targeted SNPs in the barcode were amplified successfully in the samples and controls, with a median depth of the barcode amplicons 58 (range 0–2,256 for all 407 samples/controls and amplicons, Supplementary Figure S1) and median MAF of 0.16 (range: 0.003–0.5, Supplementary Table S4). There were 2 SNPs that were not observed in the tested samples from 2018-2020, for Pf3D7_02_v3_587411 and Pf3D7_12_v3_217436 we only detected the reference allele. Two other SNPs were only observed at very low overall frequency, Pf3D7_04_v3_184289 (MAF = 0.013) and Pf3D7_09_v3_291373 (MAF = 0.012), in both cases the minor allele was only observed in Gia Lai. For 3 barcode positions, a second alternate allele was detected, but only at very low frequencies or only in controls (Supplementary Table S4). In the barcode amplicons additional SNPs were detected (per amplicon a median of 6 SNPs, with a range of 2–31 SNPs detected in one amplicon), that can be used in haplotype-based approaches.

Overall, the Pf AmpliSeq v2 Vietnam assay amplicons performed well with a median depth of 60 reads (ranging from 0-5,147 for all 407 samples/controls and amplicons). With the Pf AmpliSeq v2 Vietnam we were able to confirm the detection of validated markers of resistance in *PfK13, Pfmdr1, Pfdhfr* and *Pfdhps* in previously genotyped control samples and laboratory strains (Supplementary Table S5) with 99.7% concordance with previously genotyping results obtained with different approaches (Rovira-Vallbona et al., 2020; Rovira-Vallbona et al., 2023). There were no incorrect genotypes called, only additional alleles, indicating mixed clone infections or cross-over contamination at very few positions (in 3/1,179 genotypes, Supplementary Table S5).

There were 4 amplicons (1.5%) with very high mean DP-values (>300) (EPS15_Formin2_5, pfmrp1_2, pfdhps_11, pfhrp2_2, Supplementary Figure S1)). In addition, while there were 7 (2.6%) amplicons with low mean DP-values (<10) (amplicons pfk13_1, EPS15_Formin2_12, pfdhfr_1, EPS15_Formin2_13, pfcoronin_1, KIC7_1, pfdhps_2), these amplicons did not target any validated or candidate markers of resistance in these genes and this did not impact drug resistance genotyping results.

#### Spatial distribution of genetic markers of validated ART and partner drug resistance

**FIGURE 3:**
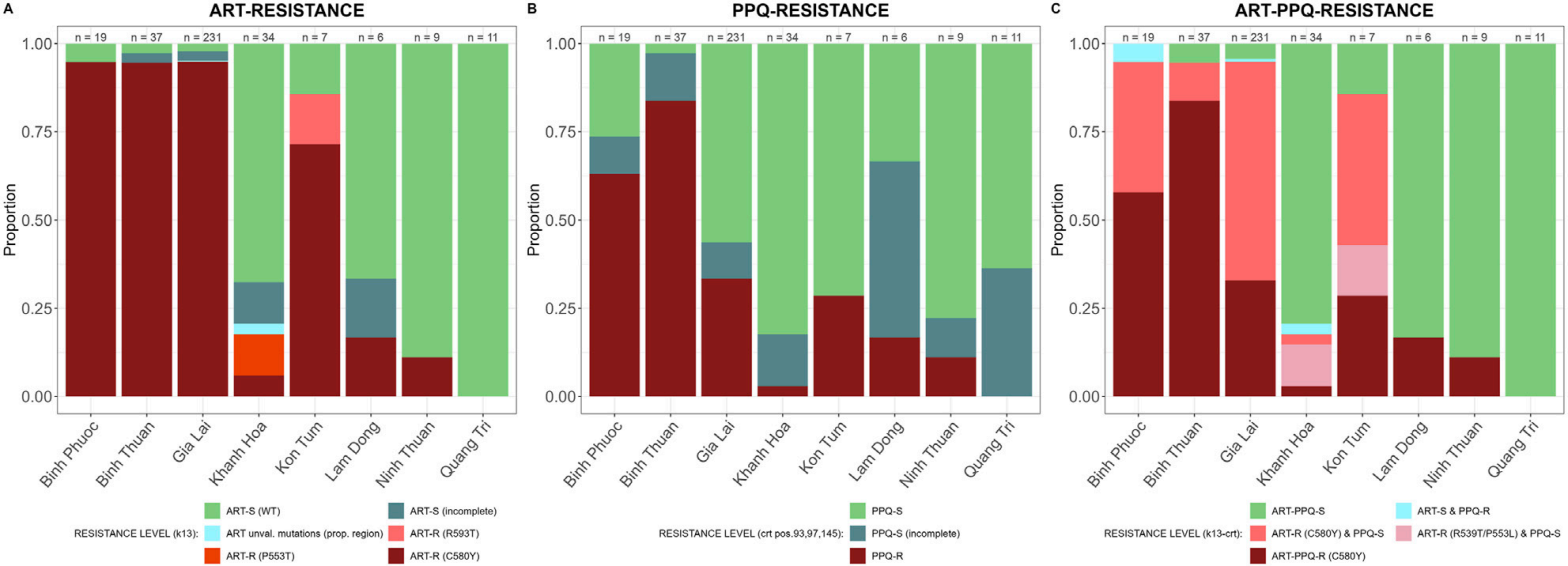
Artemisinin and partner drug resistance in 2018–2020 by province in Vietnam. Sensitive wild type (WT) in green and incomplete haplotypes in grey, resistant haplotypes in shades of red and pink. **(A)** *PfK13* validated (C580Y, R539T and P553L) and unvalidated mutations in propeller region associated with ART-R, **(B)** *Pfcrt* validated (T93S, H97Y, F145I) mutations associated with PPQ-R, **(C)** combined *PfK13* and *Pfcrt* mutations associated with ART-R and PPQ-R.

In the propeller region of the *PfK13* gene we found 9 non-synonymous SNPs of which the C580Y was the most predominant (281/354 samples) and seen in all provinces except Quang Tri (Figure 3A). ART-R parasites were observed in more than 10% of samples in all provinces, except in Quang Tri (11/11 samples in Quang Tri had wildtype *PfK13*). In addition to the WHO-validated ART-R SNP C580Y, the validated and candidate mutations P553L, I543T, R539T and P441L and 4 unvalidated SNPs were observed at low frequencies (Table 3). High proportions (>85%) of ART-R parasites were observed in Binh Phuoc, Binh Thuan, Gia Lai and Kon Tum provinces, while most samples in Khanh Hoa, Lam Dong, Ninh Thuan and Quang Tri were ART sensitive (>82%, Figure 3A). Overall, the proportion of ART-R parasites increased from 62% in 2018 to 85% and 95% in 2019 and 2020, respectively (p< 0.0001, Χ^2^ test).

**TABLE 3:** SNPs in *PfK13* propeller region detected in Vietnam.

Position in chr13	PfK13 mutation	Status	n	%	Notes
1725259	C580Y	WHO-validated	281	79.4	
1725284	N572D	Unvalidated	1	0.3	In mixed clone infection only
1725323	D559N	Unvalidated	1	0.3	In mixed clone infection only
1725340	P553L	WHO-candidate	4	1.1	
1725370	I543T	WHO-validated	1	0.3	
1725382	R539T	WHO-validated	1	0.3	
1725522	L492F	Unvalidated	1	0.3	In mixed clone infection only
1725533	N489D	Unvalidated	2	0.6	In mixed clone infection only
1725675	P441L	WHO-candidate	1	0.3	In mixed clone infection only

**FIGURE 4:**
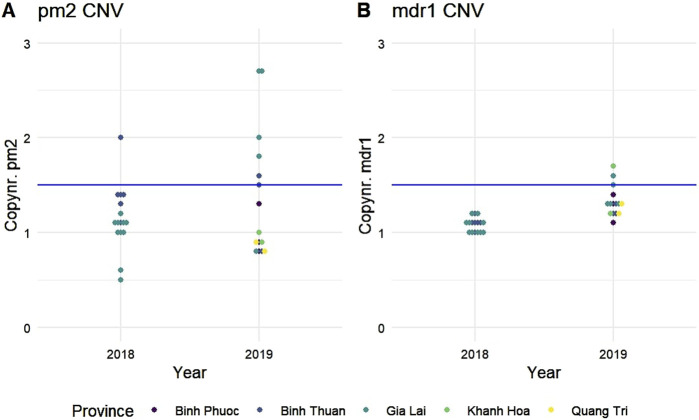
Copy number variations in **(A)** *plasmepsin 2 (Pfpm2)* and **(B)** *multi drug resistance protein 1 (Pfmdr1)*. Copy numbers were determined by qPCR on a subset of samples from 2018 to 2019. Gene amplifications were considered when the copy number was above a threshold of 1.5 (blue lines). Each dot represents a single sample and is colored by province of origin.

**FIGURE 5:**
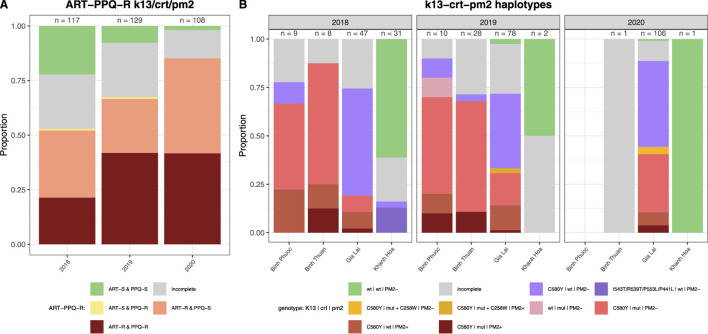
Artemisinin and piperaquine resistance by year in Vietnam. **(A)** Barplot of predicted phenotypes based on *PfK13* validated (C580Y, R539T, P553L I543T, and P441L) mutations in propeller region associated with ART-R, and *Pfcrt* (T93S, H97Y, F145I and C258W) mutations and *Pfpm2* copy number variations associated with PPQ-R. Sensitive wild type (WT) in green and incomplete haplotypes in grey, resistant haplotypes in shades of red, pink and yellow. Sample sizes for each group are indicated above the bars. **(B)** Barplot of haplotypes of *PfK13* (C580Y, R539T, P553L I543T, and P441L), *Pfcrt* (T93S, H97Y, F145I and C258W) and *Pfpm2* associated with ART-R and PPQ-R per year and province in Vietnam. (PM+: increased copy numbers; PM-: single copy). Only provinces with samples from more than 1 timepoint were included, other provinces are listed in Supplementary Figure S4. Sensitive wild type (wt) in green and incomplete haplotypes in grey, PPQ resistant haplotypes in shades of red and pink, isolates with *Pfcrt* 258W in yellow and ART-R PPQ-S haplotypes in purple. Sample sizes for each group are indicated above the bars.

PPQ resistance, mediated through WHO-validated markers T93S, H97Y, and F145I in the gene *Pfcrt,* was very high in Binh Phuoc and Binh Thuan provinces (63% and 84% respectively, Figure 3B). *Pfcrt -*mediated PPQ resistance mutations were associated with C580Y mutations in *PfK13* (123/124 samples with PPQ-resistant *crt* mutations also carried the *PfK13* C580Y mutation; Fisher’s Exact Test p< 0.0001), indicating high levels of ART and PPQ resistance in Binh Phuoc, Binh Thuan, Gia Lai and Kon Tum provinces (Figure 3C). PPQ-resistance, mediated by increased copy numbers in the *Pfpm2* gene determined by qPCR, was observed in 19% of tested samples (6/31 samples tested by qPCR, Figure 4), and increased read depth of *Pfpm2* amplicons in the Pf AmpliSeq v2 Vietnam assay was observed in 13% (41/306 samples with sufficient overall read depth for CNV analysis) (Supplementary Figure S3). Increased read depth of *Pfpm2* amplicons was observed in the provinces Binh Phuoc, Binh Thuan and Gia Lai, but only in samples that also carried C580Y *Pf*K13 mutations. In Khanh Hoa (n = 2) and Ninh Thuan (n = 1), increased read depth of *Pfpm2* amplicons was not associated with *PfK13* C580Y mutations. Taking all PPQ-R markers into account, resistance increased from 28% in 2018 to 52% and 51% in 2019 and 2020, respectively (p= 0.00017, Χ^2^ test, Table 4). This increase was driven predominantly by the increase in *Pfcrt* validated markers of PPQ resistance (Table 4). The mutation C258W in *Pfcrt*, previously reported as compensating for the fitness defect from F145I while restoring CQ resistance and PPQ sensitivity (Hagenah et al., 2024), was also seen in a few samples in Gia Lai province in 2019 (n = 2) and 2020 (n = 5), however this is not a validated marker. Nevertheless, when taking this marker into account and assuming it removes the PPQ-R mediated by the other *Pfcrt* mutations, an increase in ART&PPQ-R can be observed up to 41.8% and 41.7% in 2019 and 2020 (Figure 5A). Not all ART-R parasites are also PPQ-R, while there is an overall increase in ART-R as mentioned in the previous section. Parasites with PPQ-R *Pfcrt* mutations (with or without *Pfpm2* amplifications) were particularly abundant in Binh Phuoc and Binh Thuan province in 2018 and 2019, and increasing in Gia Lai province from 2018 to 2020 (Figure 5B).

**TABLE 4:** PPQ-resistance between 2018-2020 in Vietnam genotyped using *Pfpm2* (gene amplifications) and *Pfcrt* (T93S, H97Y and F145I) validated mutations by Pf AmpliSeq v2 Vietnam.

Year	n samples with PPQ-R by *Pfcrt* and/or *Pfpm2*	n samples/year total	% Samples with *Pfpm2-*amplified PPQ-R	% Samples with *Pfcrt* PPQ-R	% Samples with PPQ-R by *Pfcrt* and/or *Pfpm2*
2018	33	117	12%	19%	28%
2019	67	129	17%	43%	52%
2020	55	108	11%	44%	51%

#### Spatial distribution of genetic markers of CQ and SP resistance

**FIGURE 6:**
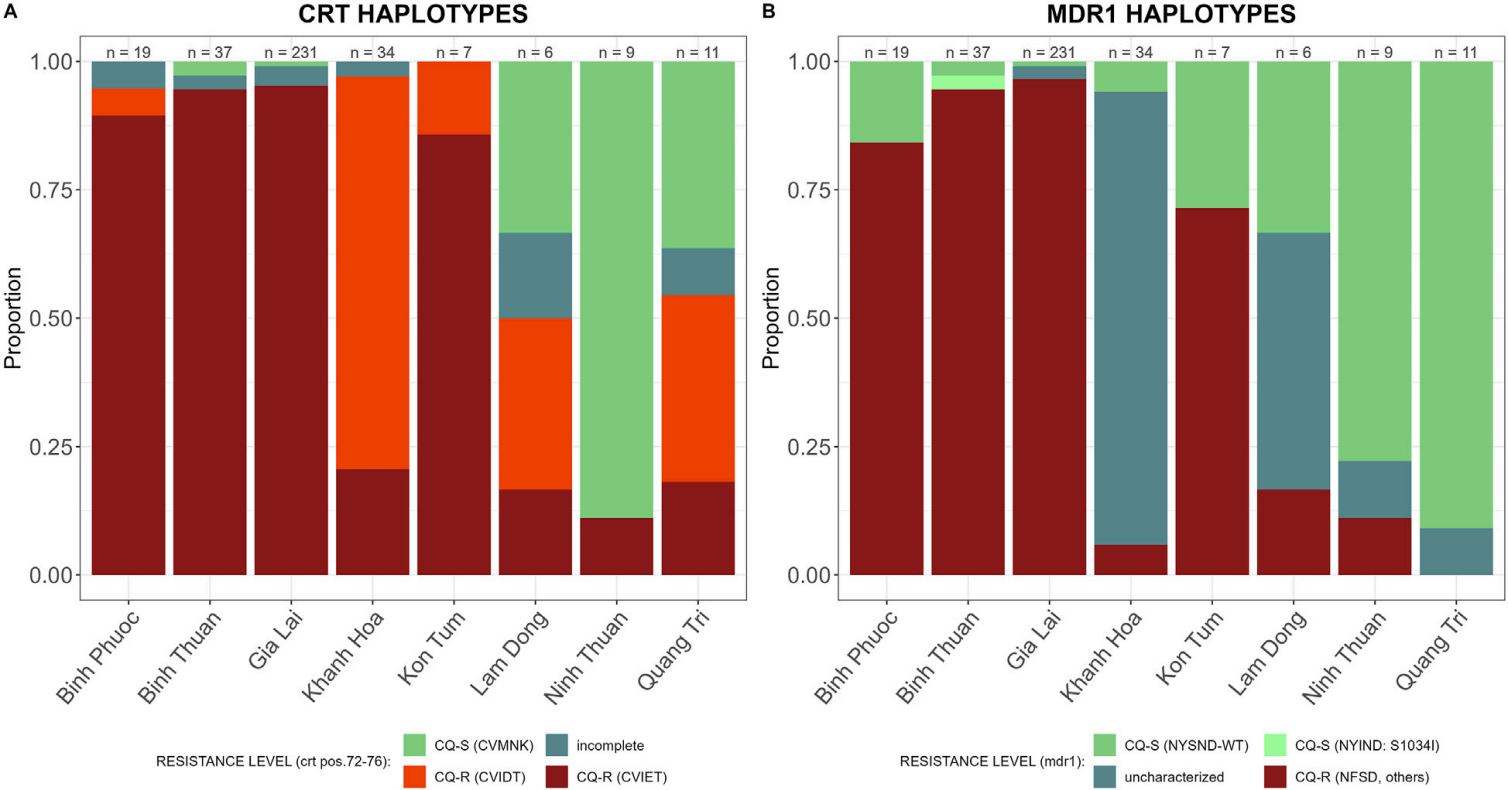
CQ resistance markers in 2018–2020 by province in Vietnam. Sensitive wild type (WT) in green and incomplete haplotypes in grey, resistant haplotypes in shades of red and pink. **(A)** *Pfcrt* (amino acid 72-76) haplotypes associated with CQ resistance, and **(B)** *Pfmdr1* (amino acid 784-1,068) haplotypes associated with CQ resistance.

CQ resistance was high in Vietnam, in all provinces except Ninh Thuan. The CQ-R haplotype CVIET was predominant in provinces with high ART-PPQ resistance, Binh Phuoc, Binh Thuan, and Gia Lai, while CQ resistant haplotype CVIDT was predominant in Khanh Hoa, Lam Dong and Quang Tri (Figure 6A). In addition, mutations in *Pfmdr1* (N86Y, Y184F, S1034C, and N1042D) intensifying *Pfcrt*-mediated CQR were common in Binh Phuoc, Binh Thuan, and Gia Lai (Figure 6B). Mutations S1034C and N1042D were seen at very low frequencies, and the predominant resistant *Pfmdr1*-haplotype was NFSND.

**FIGURE 7:**
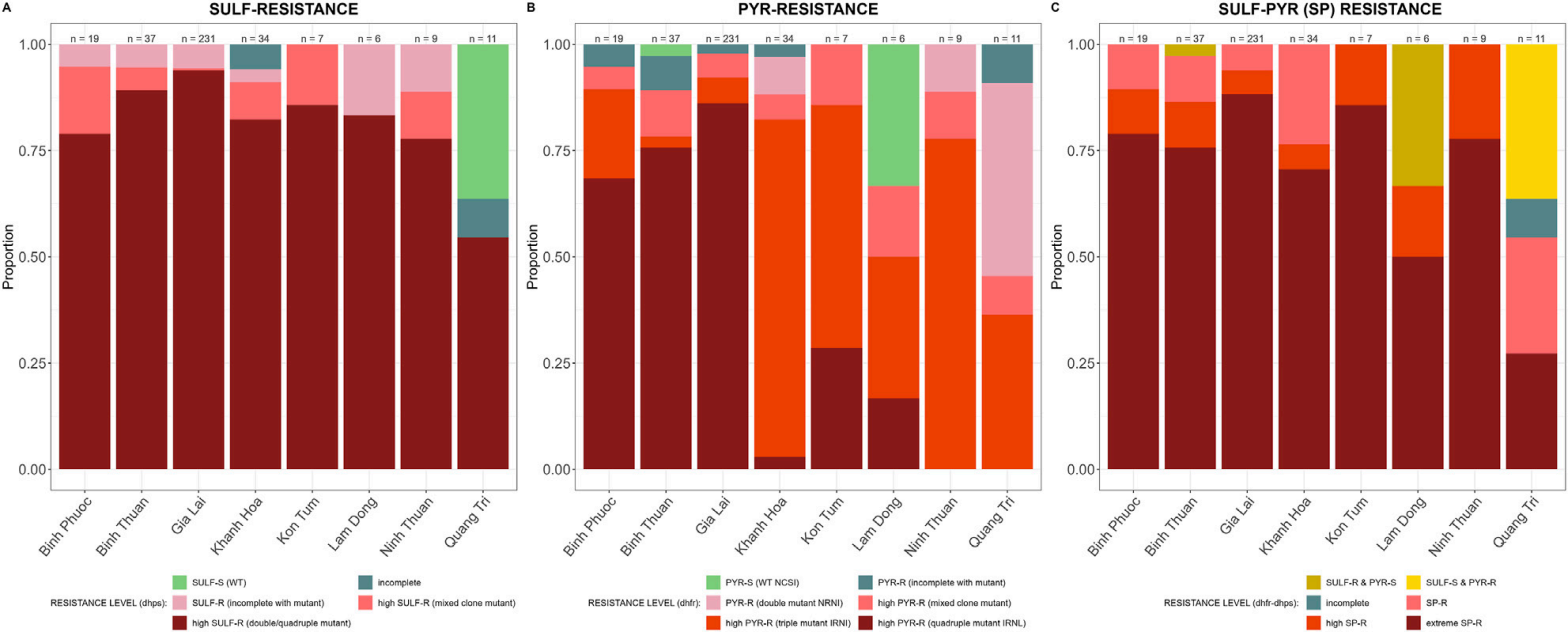
SP resistance markers by province in Vietnam. Sensitive wild type (WT) in green and incomplete haplotypes in grey, resistant haplotypes in shades of red and pink. **(A)** *Pfdhfr* (amino acid 191-1,390) haplotypes associated with sulfadoxine resistance and **(B)** *Pfdhps* (amino acid 436-613) haplotypes associated with pyrimethamine resistance and **(C)** *Pfdhfr* and *Pfdhps* haplotypes combined for SP-resistance.

SP-resistance was high, with multiple mutations in *Pfdhfr* and *Pfdhps* prevalent in all provinces (Figure 7). Some parasites in Lam Dong and Quang Tri were still susceptible to either sulfadoxine or pyrimethamine (Figure 7C).

#### Mutations in non-*Pf*K13 ART-R associated genes

The Pf AmpliSeq v2 Vietnam assay also targeted the genes *Pfubp1, Pfcoronin, Pfap-2-mu* and *PfKIC7* that are involved in the same endocytosis pathway as *PfK13* and there is some evidence of their potential association with ART susceptibility. We detected 8 samples with the ART-R-associated E1528D mutation in *Pfubp1*, all in samples from Gia Lai or Kon Tum province that also carried *PfK13* C580Y (Supplementary Table S6). The most frequent *Pfubp1* haplotype carried only the synonymous mutation R1133R.

All 3 samples with *Pf*K13 mutation P553L from Khanh Hoa carried an insert of amino acids EQKY at position 2,826 of *Pfubp1* and both S494R and F486S mutations in *Pfeps15*. In other samples, the *Pfeps15* F486S mutation was the most predominant (342/354 samples), including all samples with *PfK13* C580Y and R539T. For the *Pfcoronin* gene, the wildtype haplotype was most predominant (229/354 samples), while we also detected S183G (90/354), common in Africa and of significant interest, as it does not independently confer resistance *in vitro*, but is frequently associated with other ART-R mutations (Rosenthal and Ng, 2020; Pierreux et al., 2024), and V424I in one sample with *PfK13* C580Y. For the genes *Pfap-2-mu* and *PfKIC7*, the wild type haplotypes were also the most predominant (341/354 and 234/354 samples, respectively).

### *Pfhrp2* and *Pfhrp3* read depth

We assessed the possibility of *Pfhrp2* and *Pfhrp3* deletions by investigating the read depth. Both genes amplified very well in the assay, with general high read depth at the amplicons targeting these genes. We were able to detect *Pfhrp2* deletions in the laboratory Dd2 control strain, but only in very few study samples (n = 5) we observed a similar lack of *Pfhrp2* amplifications, indicating a deletion in this gene (Supplementary Figure S4). Similarly, *Pfhrp3* amplicons amplified well in all samples, with only very few samples where all or some of the amplicons did not amplify (n = 4). In summary, deletions in *Pfhrp2* and *Pfhrp3* were rare in Vietnam.

### Genetic diversity

Most samples (255/354, 72%) were single clone infections, based on *Fws* determined using all biallelic variants. There was no difference in the proportion of single clone infections by province (range 59%–90%, Χ^2^ p-value = 0.19). Binh Thuan province had the highest proportion of multiple clone infections (40.5%), followed by Gia Lai province (29.9%).

**FIGURE 8:**
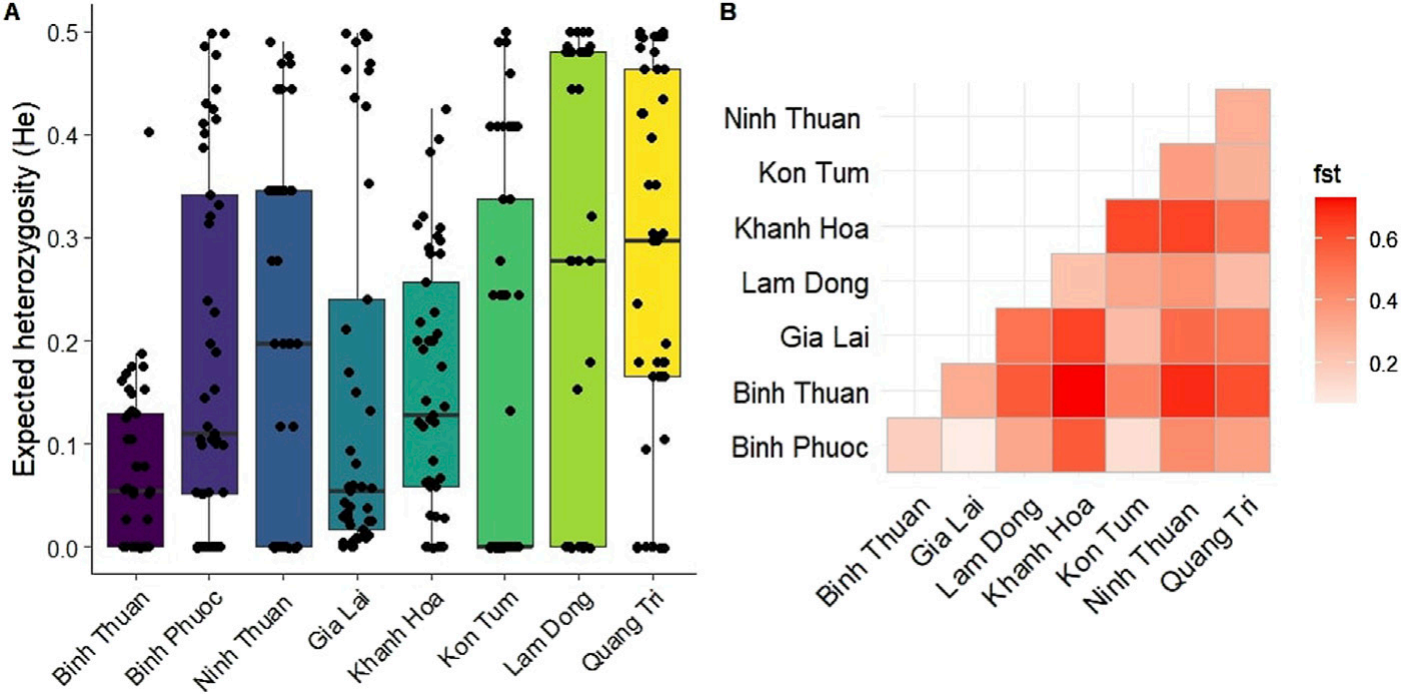
Genetic diversity and differentiation of *Plasmodium falciparum* in Vietnam. **(A)** Boxplot showing the distribution and median expected heterozygosity (*He*) at 46 SNP barcode positions for each province in Vietnam. **(B)** Heatmap of genetic differentiation (*Fst*) between the isolates from different provinces, showing high differentiation (red) in Khanh Hoa with most other provinces except Lam Dong and no differentiation (white) between Binh Phuoc and Gia Lai and Kon Tum.

Median genetic diversity (*He*) was lowest in Kon Tum (median *He* = 0), followed by Binh Thuan and Gia Lai province (median *He* = 0.054 for both, Figure 8A). Diversity was highest in Lam Dong and Quang Tri (median *He* = 0.28 and 0.30, respectively, Figure 8A), and significantly higher in Quang Tri compared to other provinces, except Ninh Thuan and Lam Dong (p< 0.05 pairwise Wilcoxon BH-corrected for multiple comparisons). While the burden of malaria (number of cases) in Gia Lai province is much higher than other provinces (Table 2), the genetic diversity was one of the lowest. Genetic differentiation (F_ST_) was low between Gia Lai province and Binh Phuoc, and Binh Phuoc and Kon Tum (Figure 8B). Parasites from Khanh Hoa showed high genetic differentiation with other provinces. Binh Thuan is different from other Coastal provinces, and more similar to Binh Phuoc and Gia Lai populations (Figure 8B).

### Population structure and connectivity

**FIGURE 9:**
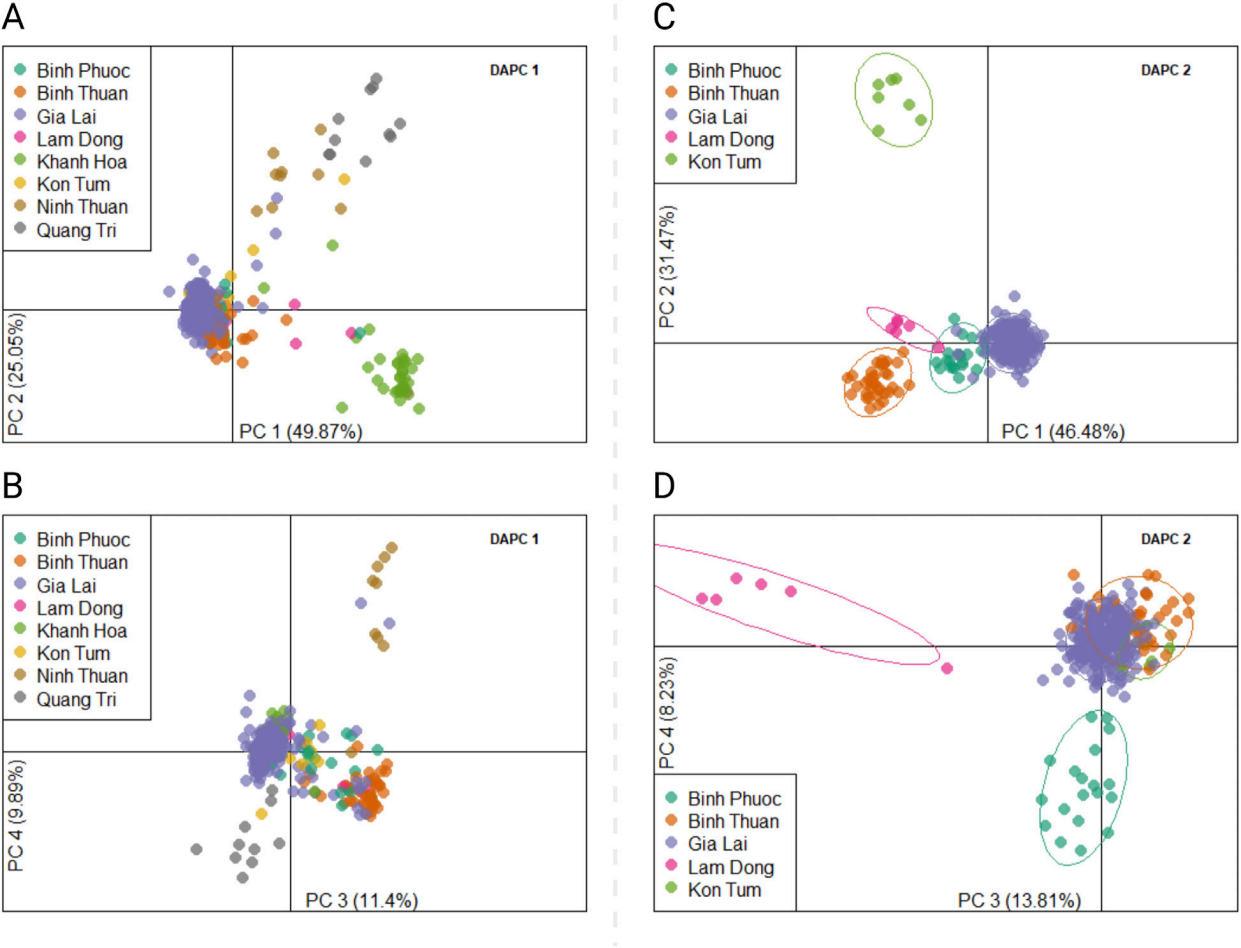
Discriminant Analysis of Principal Components (DAPC) of *Plasmodium falciparum* samples from Vietnam. DAPC calculates the discriminant components using predefined populations (here *provinces*) to group samples from the same population, while simultaneously maximizing the distance to samples from other populations. Two DAPCs were performed using all biallelic SNPs detected with the Pf AmpliSeq v2 Vietnam assay. Left, DAPC 1 **(A, B)** included all provinces and 354 samples from Vietnam, showing a scatterplot of principal component 1 and 2 **(A)**, 3 and 4 **(B)** with eigenvalues in % as the estimated contributing variance. DAPC 1 was performed with 25 principal components and 7 discriminants as determined by cross-validation. Right, DAPC 2 **(C, D)** excluded highly differentiated provinces of Khanh Hoa, Quang Tri, Ninh Thuan to increase resolution of the model to structure based on variations in the main cluster. Each dot represents one individual and colors and inertia ellipses indicate their assignment to one of the four 5 genetic clusters. DAPC 2 was performed with 140 principal components and 4 discriminants as determined by cross-validation.

Population structure between provinces in Vietnam was further explored using DAPC using all biallelic SNPs for higher resolution analysis (Figure 9). DAPC is a model in which genetic variation is partitioned into a between-group and a within-group component, and yields synthetic variables that maximize the first while minimize the second. In other words, DAPC attempts to summarize the genetic differentiation between groups, while slightly overlooking within-group variation. This is similar to PCA but uses a statistical approach to maximize between-group variation. North and South-Central Coast Provinces Khanh Hoa Ninh Thuan and Quang Tri clustered away from Central Highland provinces, Gia Lai and Kon Tum, and Binh Phuoc (Southeast region), while South Central Coast Province Binh Thuan clustered close to these provinces (Figures 9A, B). The population from Khanh Hoa clustered furthest away from other populations, except from Lam Dong, consistent with the F_ST_ results with the barcode (Figure 9A). While Ninh Thuan Province neighbors Binh Thuan, Lam Dong and Khanh Hoa provinces, the *P. falciparum* parasites in this province clustered closer to isolates from more northern provinces Quang Tri and Kon Tum along PC2 (Figure 9A), while it clustered away from all provinces in PC4 (Figure 9B). Upon closer investigation of the main cluster of Central Highland provinces and Binh Phuoc and Binh Thuan, PC1 indicated a clustering pattern from northern to southern provinces, and Kon Tum separated from the other provinces along PC2 (Figure 9C). Taken together, these results indicate that the parasite populations in the provinces of Vietnam can be differentiated at distinct levels in the DAPC analysis with marked geographical clustering of isolates in Vietnam.

**FIGURE 10:**
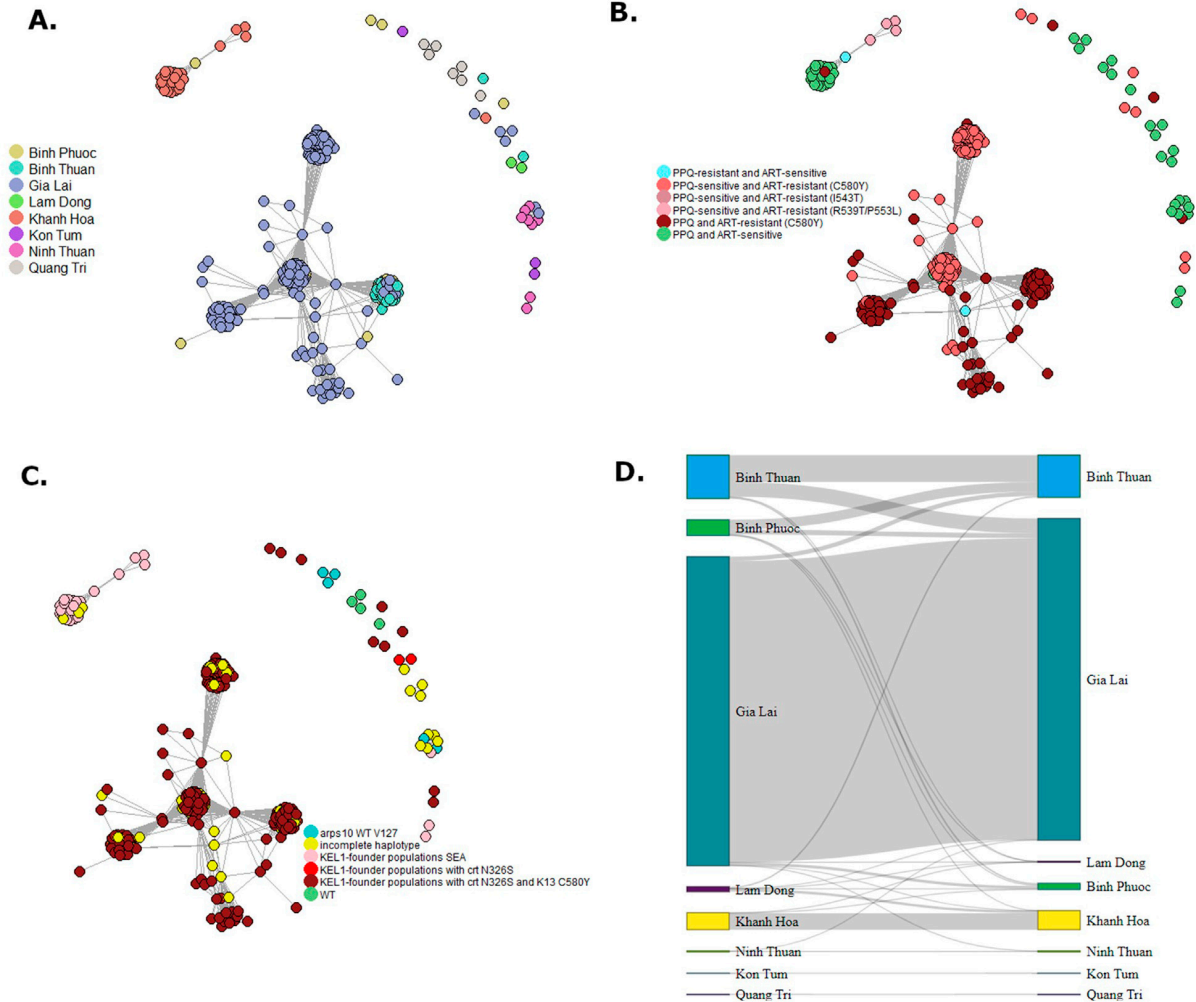
Network of cluster of *Plasmodium falciparum* parasite connectivity in Vietnam. Relatedness networks inferred by IBD between *Plasmodium falciparum* isolates **(A–C)**. Edges connecting parasite pairs indicate that >90% of their genomes descended from a common ancestor without intervening recombination. Node colors indicate the province of collection **(A)**, ART-PPQ resistance classification by *PfK13* and *Pfcrt* validated mutations **(B)** or KEL-1 lineage **(C)**. A Sankey network was made of summed pairwise IBD-sharing (>90%) between isolates between and within provinces **(D)**.

Clusters of highly related (>90% IDB-sharing) *P. falciparum* isolates were seen especially in Gia Lai and Binh Thuan province and a separate cluster in Khanh Hoa province (Figure 10A). The clonal structure of parasites in these provinces explains the observed low diversity, despite the high number of samples from these provinces. The clonal clusters in Gia Lai and Binh Thuan all belonged to the KEL1-lineage (genotyped at several loci on chromosomes 13 and 14, identifying 16 haplotypes, Supplementary Table S7) and contained the C580Y mutation in *PfK13,* and in some clusters also the PPQ-resistance conferring mutation N326S in *Pfcrt* (Figures 10B, C). Some samples from Binh Phuoc were also related to these clusters (Figure 10A). The cluster of samples from Khanh Hoa were predominantly ART- and PPQ-sensitive, while it also contained the 4 samples with the *PfK13* P553L mutation, indicating this mutation emerged in the distinct local parasite population, which also shared ancestry to the KEL1-lineage, but without mutations C580Y and N326S (Figures 10B, C). In pairwise analysis of IBD between isolates, high relatedness was seen especially between isolates within a province and less so between provinces, indicating little connectivity between the *P. falciparum* populations between provinces (Figure 10D).

## Discussion

### Findings

Vietnam is ambitiously targeting the elimination of malaria by 2030, a goal that necessitates rigorous monitoring of the disease to inform strategic interventions. The adoption of innovative tools like the AmpliSeq Pf Vietnam v2 assay, can significantly contribute to understanding the genetic diversity and drug resistance profiles of parasite populations in a region with a high level of multi-drug resistant *P. falciparum* (World Health Organization, 2022; Jacob et al., 2021). In a period of steep decline of malaria incidence in the country (2018-2020), we observed an increase in resistance to both artemisinin and its partner drug piperaquine in more provinces compared to previous studies, indicating a rapid spread. Nearly 80% of all samples analyzed (281/354) carried the C580Y mutation in the *PfK13* gene, while our earlier studies conducted in similar regions before 2018 detected validated *PfK13* mutations in approximately half the samples (Rovira-Vallbona et al., 2023). Other recent studies confirm our observations of high proportion of ART-R *PfK13* mutations, predominantly C580Y (Verschuuren et al., 2024; Thu et al., 2023). This highlights the critical need for ongoing surveillance and adaptive strategies to effectively combat malaria.

High proportions of parasites resistant to DHA-PPQ were observed in the provinces of Binh Phuoc, Binh Thuan, Gia Lai, and Kon Tum. Observations of treatment failures in several provinces in the country prompted a change in treatment guidelines by the NMCP from DHA-PPQ followed by PA as the first-line treatment for *P. falciparum* malaria in these provinces in the same period: in 2019 for Dak Nong and Binh Phuoc, followed by Gia Lai, Khanh Hoa, Phu Yen, and Dak Lak Province in 2020. In addition, we show for the first time high levels of DHA-PPQ resistance in Binh Thuan Province, observed as early as 2018. Binh Thuan province has set an ambitious goal to eliminate malaria by 2026, and the decline in reported cases suggests progress, but the spread of resistant lineages from neighboring provinces may pose challenges. Fortunately, in 2023, PA was adopted as the first-line treatment for both *P. falciparum* and *P. vivax* infections in all provinces in Vietnam, building on the success of PA in prior TES conducted in 2017–2018 and its proven efficacy in other provinces (Manh et al., 2021; Quang Bui et al., 2020; Minister of Health Vietnam, 2023). In contrast, the parasite population in Quang Tri has remained sensitive to ART, PPQ, and CQ, setting it apart from other regions. While Quang Tri province is part of the Central Highlands, and one of the provinces with the highest malaria burden in the early 2000s, malaria transmission has decreased rapidly much earlier than in other provinces in the Central Highlands, and remaining pockets are in remote areas near the border with neighboring Laos that also has little ART-R in that region (Rovira-Vallbona et al., 2023; Pongvongsa et al., 2012).

The observed *Pfpm2* gene amplifications and the persistence and spread of *Pfcrt* mutations present a complex picture of piperaquine resistance dynamics. While early studies were showing an increase in *Pfpm2* gene amplifications in Vietnam (from 3% in 2000 to 11% in 2016), our study along with other recent reports indicate a stabilization and potential decline of this marker (Rovira-Vallbona et al., 2023; Verschuuren et al., 2024). However, while a recent preprint reports the decline of resistance to both DHA and PPQ in *P. falciparum* parasites in the GMS, this interpretation is based on the decrease of *Pfpm2* gene amplifications alone and *Pfcrt* mutations associated with PPQ were not investigated (Verschuuren et al., 2024). With the extensive panel of markers in the Pf AmpliSeq v2 Vietnam assay, we detected a significant increase of PPQ resistance in 2019 and 2020 compared to previous years, primarily driven by the emerging *Pfcrt* variants, with resistance observed in over 50% of the analyzed isolates. Recent studies suggest that *Pfcrt* mutations may confer a more stable resistance phenotype with lower fitness costs than *Pfpm2* amplifications, which could explain their selection in these parasite lineages in the face of PPQ pressure (Hastings and Donnelly, 2005; Kim et al., 2019). The shift from *Pfpm2* amplifications to *Pfcrt* mutations observed here could therefore indicate an evolutionary adaptation, allowing parasites to survive better. In addition, the resistant parasite populations observed in Binh Phuoc, Binh Thuan, Gia Lai and Kon Tum provinces exhibited a clonal nature and low genetic diversity (median He ≤ 0.11), indicating that the highly resistant parasite lineage recently emerged in the KEL1-PLA1 genetic background. Nevertheless, the PPQ susceptibility profiles of parasites with only PPQ-R *Pfcrt* variants, without *Pfpm2* amplifications, requires further phenotypic characterization, as this was not possible with the DBS samples collected in this study. Previous clinical studies in Vietnam included samples with both mutations, whereas *in vitro* studies and studies in other regions have indicated that *Pfcrt* mutations alone can be sufficient to confer PPQ resistance (Van Der Pluijm et al., 2019; Manh et al., 2021; Ross et al., 2018; Boonyalai et al., 2022; Florimond et al., 2024; Silva et al., 2020).

Beyond ART and PPQ resistance, this study shows high levels of multidrug resistance, with 6/8 provinces reporting very high SP resistance levels with >70% of the parasites carrying double, triple or quadruple mutations in *Pfdhps* and *Pfdhps*. The continued use of CQ for *P. vivax* malaria treatment, together with PPQ pressure, shapes *Pfcrt* haplotypes, preventing a reversion of *P. falciparum* parasites to CQ sensitivity as seen in Africa since the introduction of ACTs (Asare et al., 2021; Laufer et al., 2010; Balikagala et al., 2020; Kateera et al., 2016). In most provinces, besides Lam Dong, Ninh Thuan and Quang Tri, CQ-R *Pfcrt* CVIET and CVIDT haplotypes were reported in >90% of the samples. Furthermore, since the change to PA as first line treatment in Gia Lai province in 2020 (and TES trials in 2017–2018), we start to observe the fitness compensatory mutation C258W in *Pfcrt,* which reportedly reverses the PPQ resistance caused by the F145I mutation while it increases CQ resistance (Hagenah et al., 2024). This C258W mutation has been shown through competitive growth assays with *pfcrt*-edited parasites to reduce the fitness defect caused by F145I, observed at increasing levels in this study (48/354 samples, with 30 samples from Binh Thuan), and has been reported to emerge in laboratory cultures and Southeast Asian isolates no longer exposed to PPQ pressure (Hagenah et al., 2024). Only few samples were observed with this mutation, and future studies including samples from the years after the treatment guideline change are necessary to assess the spread and importance of this mutation. In addition, we identified several non-*PfK13* mutations that may contribute to artemisinin resistance or enhanced parasite fitness of mutant parasites, as reported in Thailand and reflecting similar findings in African settings where additional resistance mechanisms have reduced ACT efficacy (Cerqueira et al., 2017). We detected E1528D in *Pfubp1*, associated with artemisinin resistance, in Gia Lai and Kon Tum, in parasites also carrying *PfK13* C580Y. In samples with the *PfK13* P553L mutation from Khanh Hoa, a unique insertion in *Pfubp1* was observed alongside mutations in the *Pfeps15* gene, suggesting a complex genetic landscape influencing artemisinin resistance. Further investigation into the functional significance and epidemiological relevance of these non-*PfK13* markers and in relation to the evolution of *PfK13-*mediated ART-R is needed, as it could uncover important insights into the evolving landscape of antimalarial drug resistance.

The high prevalence of multidrug resistance, including resistance to ART, PPQ, CQ, and SP, poses a significant threat to the current malaria control and elimination strategies in Vietnam. This trend underscores the need for continuous monitoring and potential revision of treatment guidelines, as evidenced by the switch to PA for uncomplicated malaria. High levels of CQ resistance in *P. falciparum* from Khanh Hoa, Binh Phuoc, and Gia Lai calls for close monitoring of CQ efficacy for *P. vivax*, especially in view of the recent outbreaks in Lai Chau and Khanh Hoa. Malaria elimination faces significant challenges, but genetic surveillance can guide interventions like mass drug administration (MDA), targeted drug administration (TDA), or enhanced vector control. For instance, Vietnam has implemented MDA in Lai Chau and plans TDA in Khanh Hoa due to the outbreaks. Molecular evaluations before and after these interventions are crucial, as past experiences indicate MDA and TDA may not offer sustained results, however this is not currently part of the strategy. Based on the high level of resistance and its continuous evolution detected in this study, it is recommended to include genetic surveillance in these outbreak scenarios.

Pyronaridine resistance in Plasmodium has been linked to high *mdr1* expression in mouse models (Kimani et al., 2014; Kimani and Shume, 2020). Although we did not investigate *mdr1* expression, we found very few samples with *mdr1* gene amplifications. While the resistance mechanism is not fully understood, pyronaridine resistance shares similarities with resistance to other quinolines (Dorn et al., 1998). Given the high resistance rates to PPQ and other quinolines in Vietnam, PA might not be ideal, and an alternative ACT may be needed for effective malaria management.

On the other hand, there was a notable lack of *Pfhrp2/3* gene deletions, which can lead to false-negative rapid diagnostic test results, consistent with other studies in the region (Molina-de La Fuente et al., 2021; Thang et al., 2022). This contrasts with certain countries in South America and the Horn of Africa, where *Pfhrp2/3* deletions are prevalent and prompted changes to RDT strategies (Molina-de La Fuente et al., 2021). The absence of such deletions in the study samples indicates that the current HRP2/3-based RDT remains suitable for the diagnosis of *P. falciparum* in the country.

*Plasmodium falciparum* populations in Vietnam are becoming more isolated through time, as indicated by the high proportion of clonal populations, with a high degree of geographical clustering by province, indicating limited connectivity between parasite populations across different provinces. These distinct, localized subpopulations may have evolved independently in response to an interplay of local environmental factors, epidemiological patterns, and population movements. Interestingly, the Central Highland provinces, and especially Gia Lai province, is the region with the highest residual burden of malaria in Vietnam, while it had the lowest diversity and very clonal population. This is likely due to the drug resistance profile of the parasites in this province, combined with strong selection pressure exerted/posed by the targeted efforts from the NCMP to reduce malaria in this province. The observed geographical isolation of these subpopulations is important for malaria elimination efforts, as it can promote the development and spread of region-specific drug resistance profiles that may require tailored intervention strategies. The NMCP efforts in Vietnam are organized however on a provincial level, and this localized approach might be one of the factors contributing to the success of the country in reducing its burden. Similar trends of geographically isolated parasite populations have been reported in Vietnam and the Greater Mekong Subregion, indicating a regional pattern of fragmented malaria transmission (Verschuuren et al., 2024; Cui et al., 2012).

### Strengths and limitations

The comprehensive approach of this study provides a robust framework for understanding drug resistance and its evolution. By examining a broad panel of full-length resistance genes spanning multiple drug classes and a wide range of SNPs, it provided a high-resolution perspective on the genetic diversity and resistance evolution in this country. This resulted in valuable insights that can inform local policy and decision makers to improve treatment and intervention strategies. However, the limited geographic scope and sample size highlights the need for broader surveillance in more malaria endemic provinces to capture the full spectrum of malaria resistance in Vietnam, including for example, the sites of recent outbreaks. Low incidence in some provinces made it difficult to collect large numbers of samples for statistical analysis. However, the few samples that we did include often represented >10% of the reported malaria cases in the province, making it still a meaningful assessment to inform NMCPs. In contrast, TES in these settings would not be feasible due to the low incidence, demonstrating the added value of molecular surveillance in a pre-elimination scenario. However, for genetic surveillance data to be actionable, these results need to be produced much faster and become integrated with routine epidemiological and entomological monitoring, to allow for a comprehensive understanding of malaria transmission and control. Moreover, a centralized database for sharing genetic surveillance data would facilitate coordinated responses and efficient resource allocation. Enhancing collaboration with local health authorities and communities is essential for a more representative data collection and swift outbreak response, supporting the country’s efforts towards malaria elimination.

The coordinated implementation of various approaches, including targeted drug administration, vector control measures, active surveillance and community engagement strategies, is crucial for malaria elimination efforts. However, financial constraints and changes in procurement regulations have made it challenging to obtain the necessary chemicals, antimalarial drugs, and to mobilize sufficient resources for effective implementation of these interventions and surveillance activities. This lack of coordination and resource availability can hinder the synchronization of the various control measures, thereby increasing the risk of the further spread and proliferation of drug-resistant malaria strains across the region. Addressing these systemic challenges through increased and sustained funding, streamlined procurement processes, and a comprehensive, multi-faceted strategy is essential to effectively combat the escalating threat of drug-resistant malaria and achieve the goal of malaria elimination in Vietnam.

## Conclusion

In conclusion, this study provided a comprehensive assessment of the genetic markers associated with multi-drug resistance in *P. falciparum* isolates from 8 provinces in Vietnam between 2018-2020 with the AmpliSeq Pf Vietnam v2 assay. High levels of artemisinin resistance are seen in the residual clonal parasite population as the malaria burden is decreasing while the country works towards the ambitious goal of eliminating malaria by 2030. The interventions have resulted in clonal and isolated pockets of parasites, characterized by low genetic diversity and limited genetic exchange between them. This study highlights the critical need for ongoing surveillance and adaptive strategies to effectively eliminate malaria as swiftly as possible, especially considering the potential similarities in resistance mechanisms for quinolines and pyronadine, the partner drug in the current first line ACT.

## Data Availability

The datasets presented in this study can be found in online repositories. The names of the repository/repositories and accession number(s) can be found in the article/Supplementary Material.
