# Supplementary material for: Genetic surveillance shows spread of ACT resistance during period of malaria decline in Vietnam (2018-2020)
Source: Front Genet. 2024 Dec 2;15:1478706. doi: 10.3389/fgene.2024.1478706 (PMC11646998; doi:10.3389/fgene.2024.1478706)
Supplement: Supplementary file 8 [file Image1.pdf]

Supplementary figures

A.

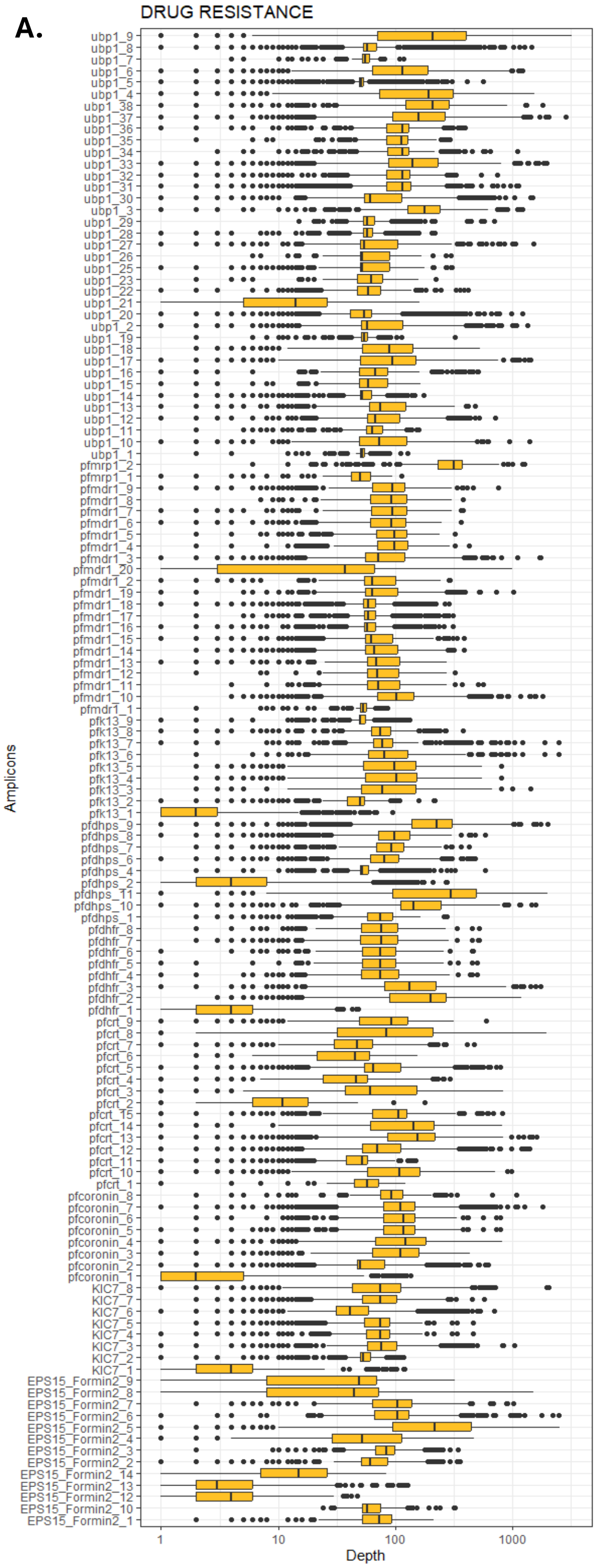

Supplementary figure 1A. Distribution of depth of coverage for amplicons for resistance associated genes in the AmpliSeq Pf Vietnam v2 assay

B.

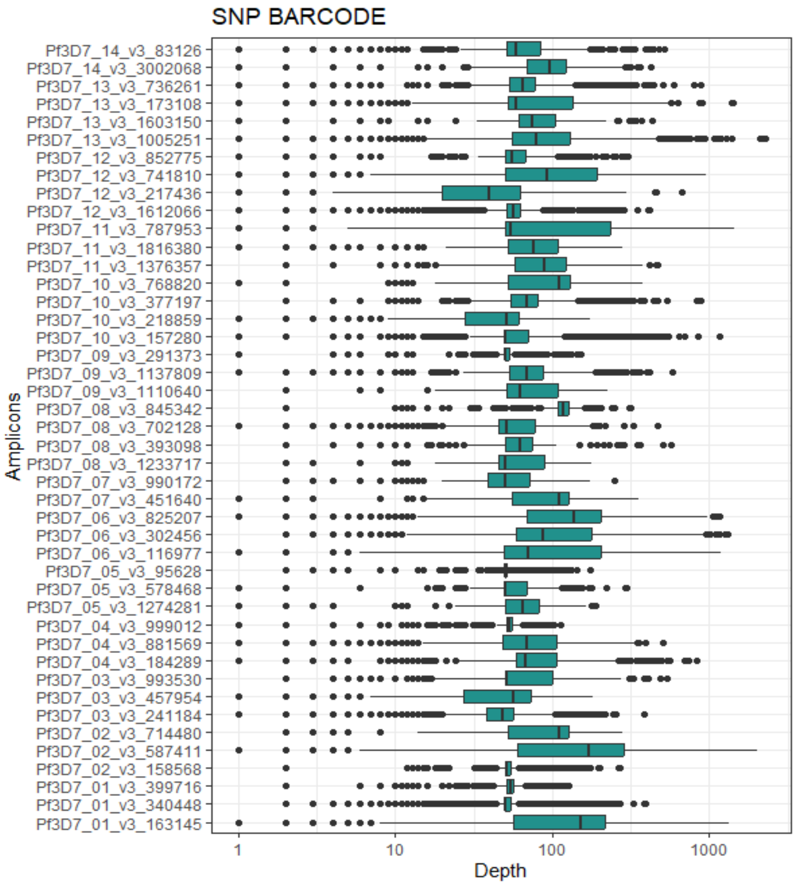

C.

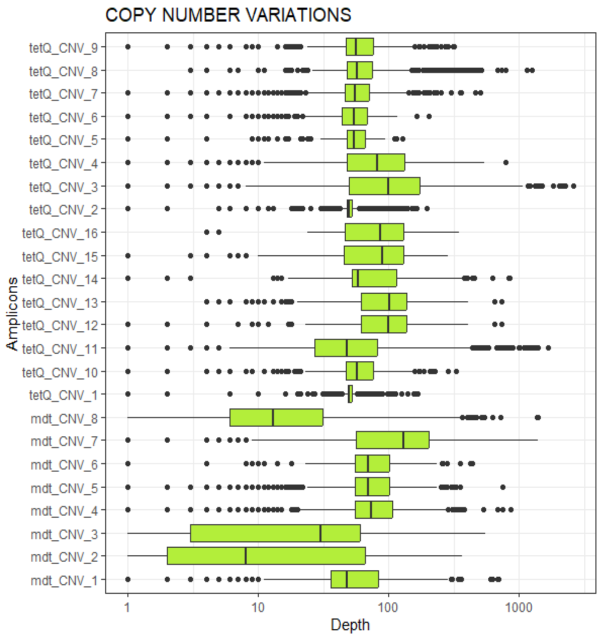

Supplementary figure 1. (B) Distribution of depth of coverage for Vietnam SNP barcode amplicons in the AmpliSeq Pf Vietnam v2 assay. And (C) amplicons for copy number variations in the AmpliSeq Pf Vietnam v2 assay.

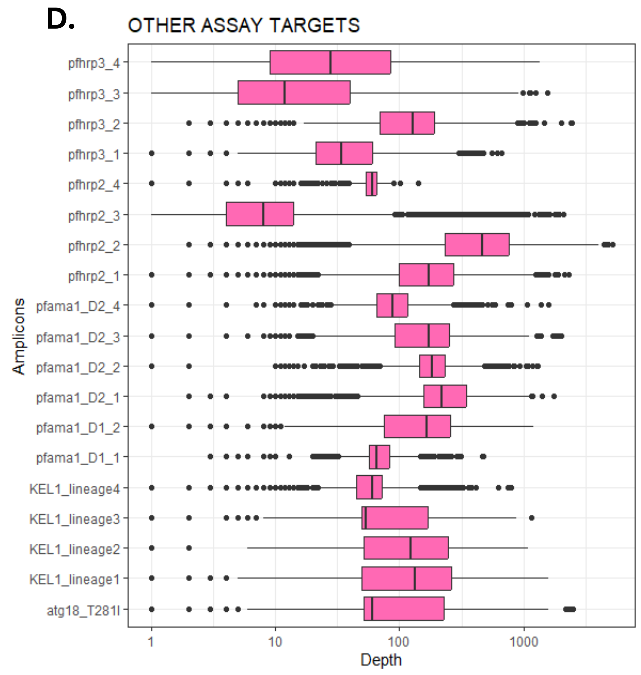

Supplementary figure 1D. Distribution of depth of coverage for remaining amplicons in the AmpliSeq Pf Vietnam v2 assay

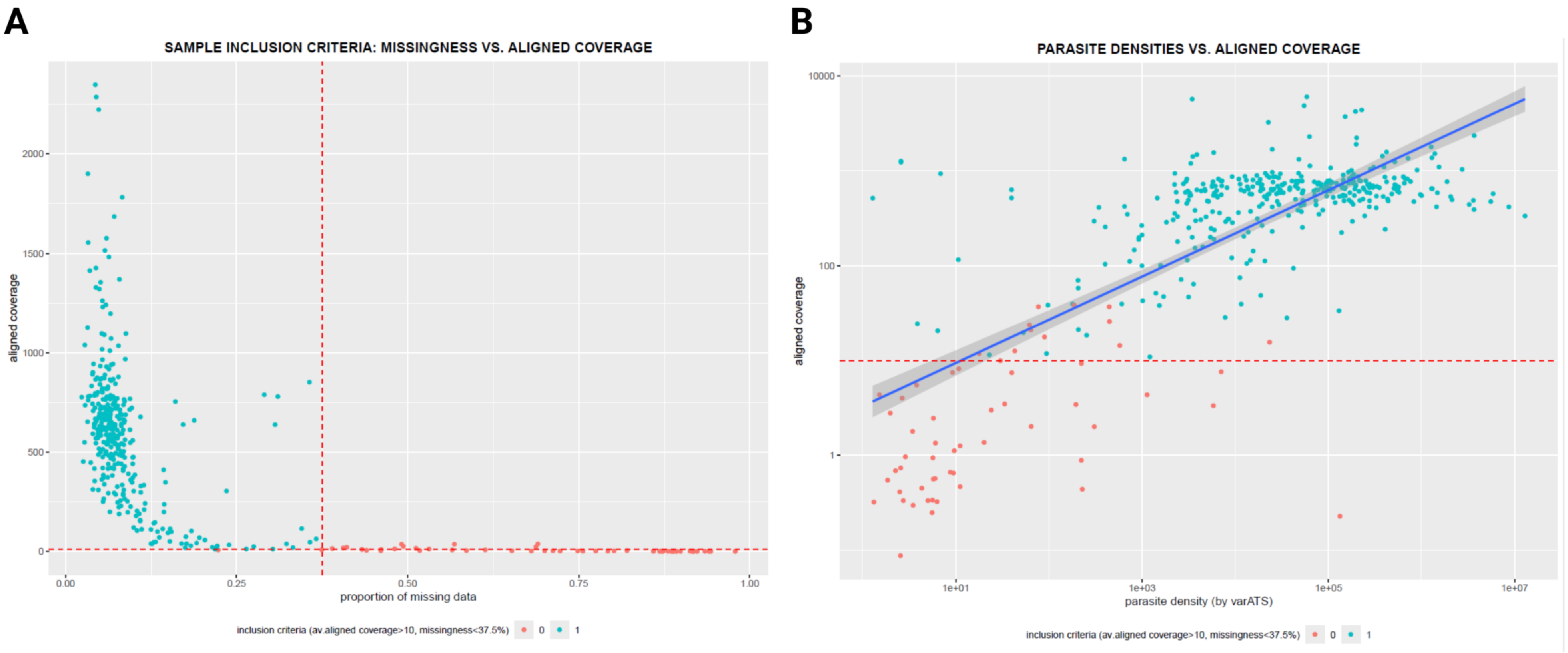

Supplementary figure 2. Depth of coverage in the AmpliSeq Pf Vietnam v2 assay *vs.* proportion of genotypes missing per sample (A), and depth of coverage *vs.* parasite density (parasites per µL) by VarATS qPCR (B).

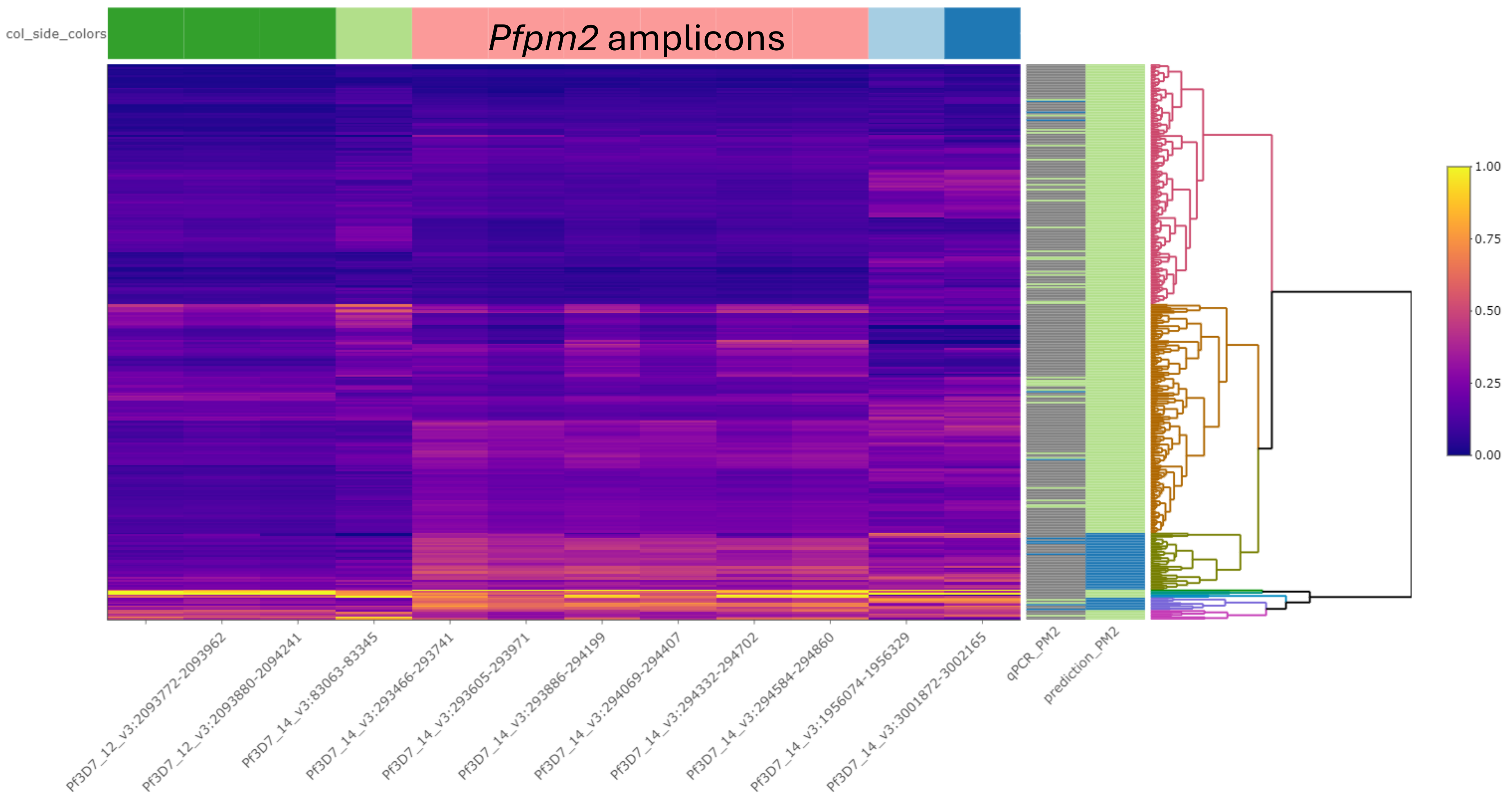

Supplementary figure 3. Heatmap of normalized read depth from BAM files of *Pfpm2* and reference amplicons (columns) of 354 samples (rows). In the annotation rows to the left is indicated 1) *Pfpm2* amplifications as determined by qPCR, and 2) prediction of *Pfpm2* amplifications based on the hierarchical clustering. Clusters are determined using hierarchical clustering using the heatmaply package in R, based on Euclidean distances between read depth profiles of samples using the complete clustering method. Resulting clusters are colored in the tree to the left.

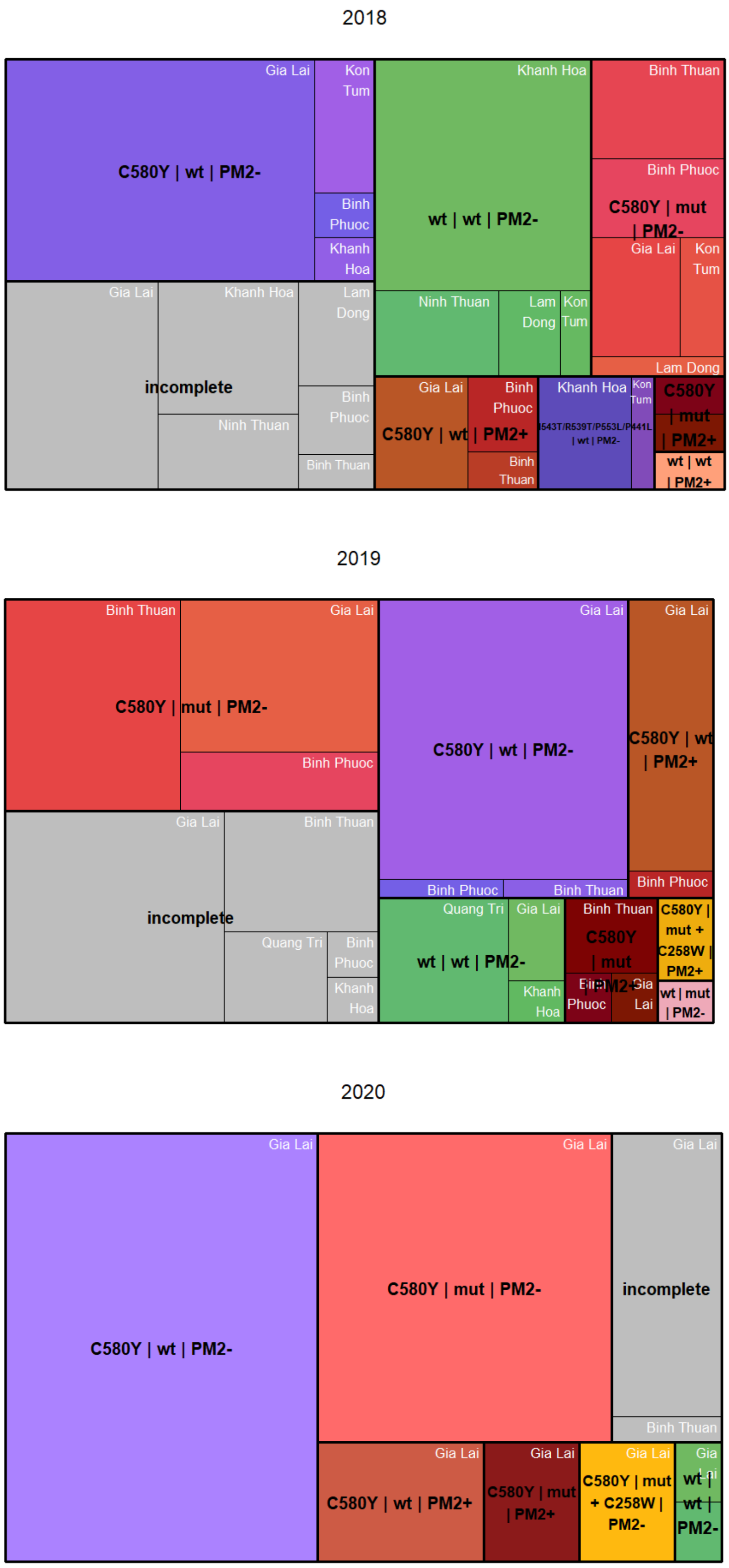

Supplementary figure 4. Treemaps showing the distribution of haplotypes of *Pfk13*, *Pfcrt* and *Pfpm2* in Vietnam by year and province. Haplotypes were constructed of genotypes in *PfK13* (loci C580Y, R539T, P553L I543T, and P441L), *Pfcrt* (loci T93S, H97Y, F145I and C258W) and *Pfpm2* copy number variations (PM+ : increased copy numbers; PM- : single copy). These genotypes have been associated with ART-R and PPQ-R.

A.

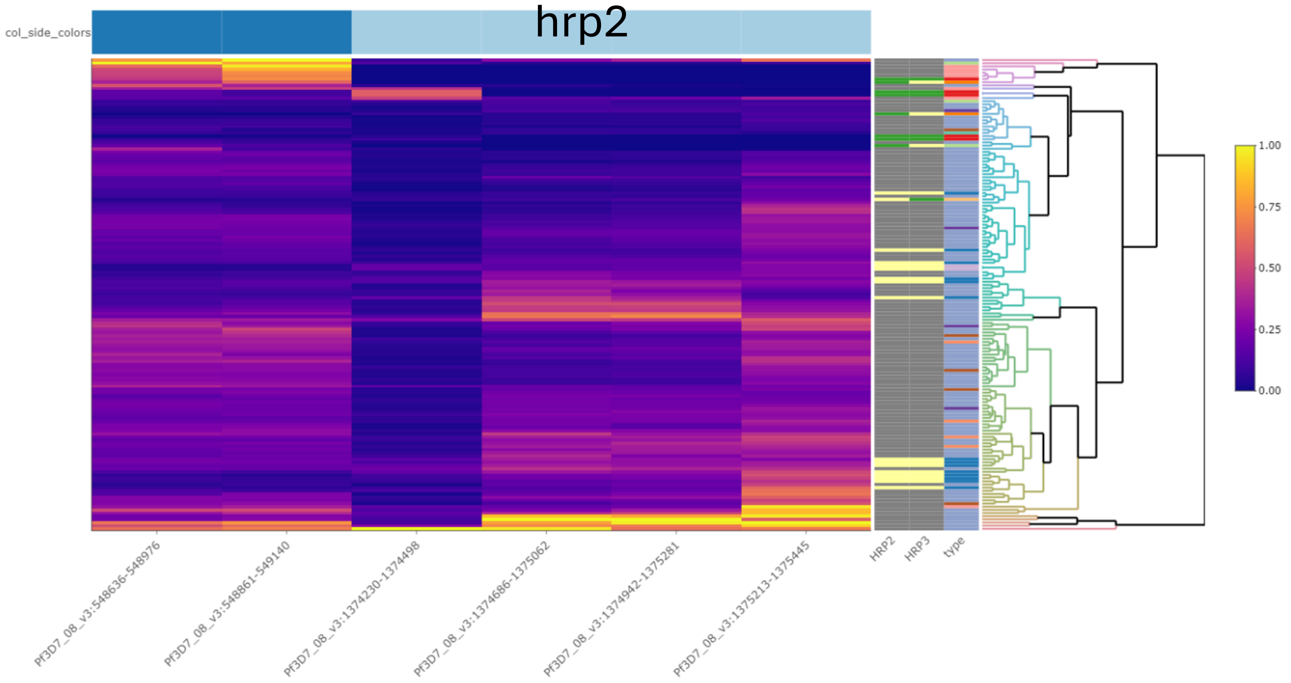

B.

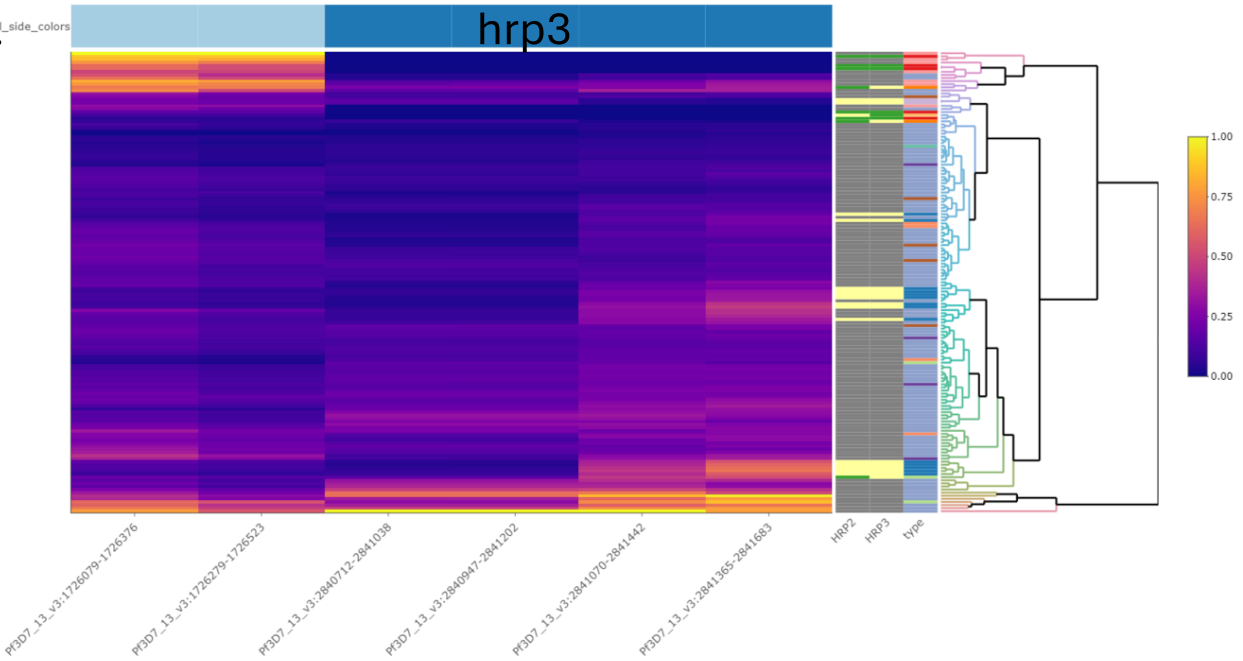

Supplementary figure 4. Heatmap of normalized read depth from BAM files of *Pfhrp2* (A) and *Pfhrp3* (B) and reference amplicons (columns) of 354 samples (rows). In the annotation rows to the left is indicated 1) *hrp2* deletions as determined by PCR or from control strains (3D7 or Dd2), yellow = gene present, green = gene deleted, and 2) *hrp3* deletions as determined by PCR or from control strains (3D7 or Dd2), yellow = gene present, green = gene deleted, and 3) type of sample (lightblue = sample from Vietnam with unknown *hrp2/hrp3* status; darkblue = 3D7; red & pinks= previously typed control sample from Peru with and without *hrp2* and *hrp* deletions (Kattenberg et al. 2023, https://doi.org/10.1128/spectrum.00960-22); lightgreen = laboratory control strains (including Dd2).
